# *seco*-Briarellinone and Briarellin S, Two New Eunicellin-Based Diterpenoids from the Panamanian Octocoral *Briareum asbestinum*

**DOI:** 10.3390/md10112608

**Published:** 2012-11-21

**Authors:** José Félix Gómez-Reyes, Ana Salazar, Héctor M. Guzmán, Yisett González, Patricia L. Fernández, Armando Ariza-Castolo, Marcelino Gutiérrez

**Affiliations:** 1 Center for Drug Discovery and Biodiversity, Institute for Scientific Research and Technology Services (INDICASAT), Clayton, City of Knowledge, P.O. Box 0843-01103, Panama; Email: jfgomez@cinvestav.mx (J.F.G.-R.); ansal2181@yahoo.com (A.S.); 2 Department of Chemistry, Center for Research and Advanced Studies of the National Polytechnic Institute (CINVESTAV-IPN), P.O. Box 14-740, 07000, F.D., Mexico; Email: aariza@cinvestav.mx; 3 Smithsonian Tropical Research Institute, Balboa, Ancon, P.O. Box 0843-03092, Panama; Email: guzmanh@si.edu; 4 Center for Molecular and Cellular Biology of Diseases, Institute for Scientific Research and Technology Services (INDICASAT), Clayton, City of Knowledge, P.O. Box 0843-01103, Panama; Email: yisettgonzalez@gmail.com (Y.G.); patryllanes@gmail.com (P.L.F.)

**Keywords:** *Briareum asbestinum*, *seco*-briarellins, briarellin diterpenes, *seco*-asbestinin diterpenes, anti-inflammatory properties

## Abstract

Two new eunicellin-based diterpenes, *seco*-briarellinone (**1**) and briarellin S (**2**), and a known *seco*-asbestinin (**3**) have been isolated from the methanolic extract of the common octocoral *Briareum asbestinum *collected in Bocas del Toro, Caribbean of Panama. The structures and relative stereochemistry of the compounds were defined using extensive spectroscopic analysis including 1D, 2D-nuclear magnetic resonance (NMR) and high-resolution mass spectrometry (HRMS). compounds **1 **and **2** displayed anti-inflammatory properties inhibiting nitric oxide (NO) production induced by lipopolisacharide (LPS) in macrophages with an Inhibitory concentration 50% (IC_50_) of 4.7 μM and 20.3 μM, respectively. This is the first report of briarellin diterpenes containing a ketone group at C-12.

## 1. Introduction

The briarellins are a family of tetracyclic diterpenes structurally derived from the eunicellin skeleton [1,2,3,4,5,6,7]. To date about 27 briarellins have been isolated and described from octocorals of the genus *Briareum *and *Pachyclavularia*. A smaller group of eunicellin-related diterpenoids, composed by only three members, is known as *seco*-briarellins [1,4,7]. *seco*-Briarellins have a nine atom heterocycle across C-2/C-9 open at the C-6/C-7 bond as a main difference to the briarellin skeleton. The biological potential of briarellins and *seco*-briarellins has not been explored extensively, however some members of these groups displayed moderate antimalarial activity and cytotoxicity [8].

As a part of a drug discovery program at the Institute for Scientific Research and Technology Services (INDICASAT), we are exploring the marine invertebrates’ diversity of Panama as source of novel anti-inflammatory compounds. Herein we report the isolation and structural determination of the new diterpenes *seco*-briarellinone (**1**) and briarellin S (**2**) and the known *seco*-asbestinin (**3**) isolated from the gorgonian octocoral *Briareum asbestinum* (Pallas) collected in Bocas del Toro, Panama. The coral is abundant and widely distributed in shallow reef environments along the Caribbean coast. compounds **1 **and **2** showed anti-inflammatory activity and are the first briarellin-type diterpenoids containing a keto group at position 12.

## 2. Results and Discussion

**Figure 1:**
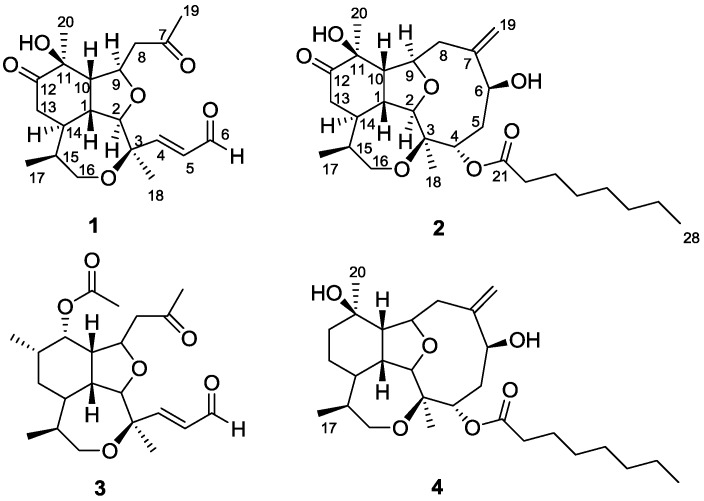
Structures of compounds **1**–**4**.

The octocoral *Briareum asbestinum* was collected by hand, using scuba in the Bastimentos National Park on the Caribbean side of Panama at a depth of 10 m. The sample was extracted with methanol-dichloromethane and the crude extract was fractionated using two subsequent silica gel columns followed by high performance liquid chromatography (HPLC) purification to yield compounds **1**–**3** (Figure 1). 

High-resolution electrospray ionization-time-of-flight-mass spectrometry (HRESI-TOF-MS) spectrum of compound **1** showed a pseudo-molecular ion peak [M + Na]^+^ at *m/z* 387.1775 corresponding with the molecular formula C_20_H_28_O_6_Na. ^13^C-NMR and heteronuclear multiple bond correlation (HMBC) spectra (Table 1) showed resonances for 20 carbon atoms, while distortion-less enhancement by polarization transfer (DEPT) and multiplicity edited heteronuclear single quantum coherence (HSQC) experiments revealed four quaternary carbons, nine methines, three methylenes and four methyl groups (Table 1). ^13^C-NMR chemical shifts revealed the presence of two ketones (*δ*_C_ 213.4, 205.8); one aldehyde (*δ*_C_ 193.9) and one double bond (*δ*_C_ 158.6, 131.0). Additionally, resonances for five carbons bearing oxygen were assigned as two methines (*δ*_C_ 76.5, 90.7), one methylene (*δ*_C_ 67.6) and two quaternary carbons (*δ*_C_ 75.4, 77.6). Seven degrees of unsaturation were inferred from the molecular formula: four accounted for three carbonyl groups and one double bond, therefore compound **1** has three rings. The fact that compound **1** possessed 20 carbon atoms and that the source organism was a coral strongly suggested that compound **1** was a diterpene.

**Table 1:** Nuclear magnetic resonance (NMR) Spectroscopic Data (CDCl_3_) for *seco*-briarellinone (**1**).

Position	*δ*_C_, mult. ^a^	*δ*_H_, mult. *J* in Hz ^b^	HMBC ^c^	COSY
1	39.3 (CH)	2.26, dt, 2.0, 9.3		2, 10, 14
2	90.7 (CH)	3.87, d, 9.3	1, 4	1
3	77.6 (C)	-		
4	158.6 (CH)	6.85, d, 15.6	3, 6	5
5	131.0 (CH)	6.42, dd, 8.3, 15.6	3, 6	4, 6
6	193.9 (CH)	9.60, d, 8.3	5	5
7	205.8 (C) ^c^	-		
8	49.4 (CH_2_)	2.79, dd, 16.3, 3.5	7, 9	9
8		2.69, dd, 16.3, 6.1		
9	76.5 (CH)	4.68, dt, 3.5, 6.1		8, 10
10	51.0 (CH)	2.08, m	11	1, 9
11	75.4 (C)	-		
12	213.4 (C) ^c^	-		
13α	38.4 (CH_2_)	2.48, dd, 14.2, 4.5	1, 12	14
13β		2.37, m		
14	37.9 (CH)	2.36, m		1, 13α, 15
15	35.7 (CH)	1.78, m		14, 16, 17
16α	67.6 (CH_2_)	3.66, dd, 3.4, 13.2	3, 17	15
16β		3.81, d, 13.2		
17	10.9 (CH_3_)	1.00, d, 6.8	14, 15, 16	15
18	24.3 (CH_3_)	1.47, s	2, 3, 4	
19	30.6 (CH_3_)	2.20, s	7, 8	
20	23.7 (CH_3_)	1.45, s	10, 11, 12	

^a^ Chemical shift values are in ppm relative CDCl_3_ residual signals; ^b^* δ* values were obtained by the assistance of the HSQC-edited spectrum; ^c^ The *δ*_C_ values were obtained by means of the HMBC correlations.

**Figure 2:**
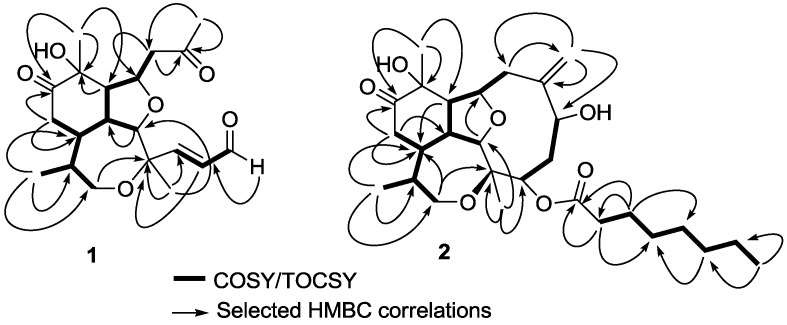
Correlation spectroscopy (COSY) or total correlation spectroscopy (TOCSY) and heteronuclear multiple bond correlation (HMBC) correlations of compounds **1** and **2**.

Inspection of the ^1^H-NMR spectrum of compound **1** showed resonances for an aldehyde proton (*δ*_H_ 9.60, d, *J* = 8.3 Hz, H-6) coupled with an olefinic proton (*δ*_H_ 6.42, dd, *J *= 8.3, 15.6 Hz, H-5). Proton H-5 was also coupled with H-4 whose resonance appeared at *δ*_H_ 6.85 ppm (d, *J* = 15.6 Hz) forming a α,β-unsaturated aldehyde. The spin system comprised by H-4, H-5, H-6 was confirmed by crossed correlations observed in correlation spectroscopy (COSY) experiments (Figure 2). The geometry of the C-4/C-5 double bond was assigned as *trans* on the basis of the coupling constant observed for protons H-4 and H-5 (*J*_H4,H5_ = 15.6 Hz) [1].

Additionally, signals for four methyl groups were observed: one secondary at *δ*_H_ 1.00 (d, *J* = 6.8 Hz), and three tertiary attached to quaternary carbons bearing oxygen (*δ*_H_ 1.45, *δ*_C_ 75.4; *δ*_H_ 1.47, *δ*_C_ 77.6; *δ*_H_ 2.20, *δ*_C_ 205.8). Remaining protons including, two α-ketone diastereotopic methylenes (*δ*_H_ 2.37, 2.47; 2.79, 2.69), one diastereotopic oxymethylene (*δ*_H_ 3.81, 3.66), and six methines (*δ*_H_ 4.68, 2.08, 2.26, 3.87, 2.36, 1.78), were all connected forming a long and branched spin system using COSY and total correlation spectroscopy (TOCSY) experiments (Figure 2). Positioning and connectivity of all functional groups and spin systems described above were carried out using ^2,3^*J* HMBC experiments (Figure 2). Thus, the α,β-unsaturated aldehyde was attached to C-3 based on a correlation observed for H_3_-18 to C-4. Also ^2^*J* HMBC correlations observed for methylene (H_2_-8) and methyl (H_3_-19) protons to C-7 allowed the assignment of an acetonyl moiety. This acetonyl group was connected to C-9 using COSY (H_2_-8 to H-9) and ^2^*J* HMBC (H_2_-8 to C-9) correlations. Methyl protons H_3_-17, H_3_-18 and H_3_-20 gave strong HMBC correlations with their adjacent carbons (Figure 2) supporting the assignment of the tricyclic scaffold of compound **1**. Finally, positioning of the keto group on C-12 was based on HMBC correlations observed for H_3_-20 and H_2_-13 to C-12 (Figure 2). Therefore the assignment of the planar structure of compound **1** was established as a novel *seco*-briarellin diterpene depicted in Figure 1. Comparison of the NMR data of compound **1** with previously reported *seco*-briarellins [1,4,7] evidenced the presence of the keto group at C-12 as a unique structural feature for compound **1**.

**Figure 3:**
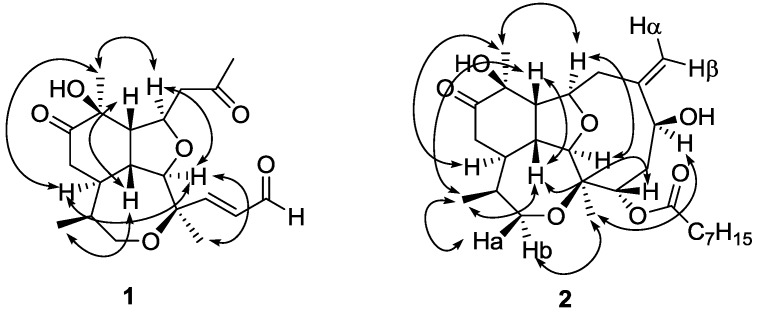
NOE correlations of compounds **1** and **2**.

The relative stereochemistry of compound **1** was assigned by double pulsed field gradient spin echo-nuclear Overhauser effect (DPFGSE-NOE) experiments (Figure 3). A strong enhancement of protons H-2 and H_3_-20 was observed when methine H-9 was irradiated, whereas irradiation of H-2 strongly enhanced the signals of H-9, H-14 and H_3_-18. Also proton H-14 was enhanced when H-15 was irradiated, while irradiation of H_3_-20 enhanced protons H-9 and H-14. All these protons (H_3_-20, H-9, H-2 and H-14) were assigned arbitrarily as having α-configuration. On the other hand, irradiation of proton H-1 produced the enhancement of both, H-10 and H_3_-17, and these protons were assigned as having β-configuration.

HRESI-TOF-MS spectrum of compound **2** showed a pseudo-molecular ion peak [M + Na]^+^ at *m/z* 515.2975 corresponding with the molecular formula C_28_H_44_O_7_Na. ^13^C-NMR and HMBC data (Table 2) showed resonances for 28 carbon atoms. DEPT and multiplicity edited HSQC experiments evidenced the presence of five quaternary carbons, eight methines, eleven methylenes and four methyl groups (Table 2). Seven degrees of unsaturation were inferred from the molecular formula: two carbonyl groups assigned as a ketone and ester (*δ*_C_ 214.6 and 175.6) and an exocyclic double bond (*δ*_C_ 152.8 and 115.8) accounted for three unsaturations, therefore compound **2** has four rings.

**Table 2:** NMR spectroscopic data (CDCl_3_) for briarellin S (**2**).

Position	*δ*_C_, mult. ^a^	*δ*_H_, mult. (*J* in Hz) ^b^	HMBC ^c^	COSY
1	38.5 (CH)	2.80, m		2, 10, 14
2	92.9 (CH)	3.97, d, 8.8	3, 4, 9, 14, 18	1
3	76.6 (C)	-		
4	71.4 (CH)	5.08, br s		5
5	36.4 (CH_2_) ^b^	1.71, br d ^b^		4
6	73.8 (CH)	4.18, br s		
7	152.8 (C) ^c^	-		
8	39.5 (CH_2_)	2.33, m	19	9
9	81.5 (CH)	4.68, br s		8, 10
10	49.1 (CH)	2.54, m	14	1, 9
11	75.7 (C)	-		
12	214.6 (C)	-		
13α	39.4 (CH_2_)	2.38, m ^b^	1, 12, 14	14
13β		2.38, m ^b^		
14	39.3 (CH)	2.23, m ^b^		1, 13α, 15
15	35.5 (CH)	1.66, m ^b^		14, 16α, 17
16α	66.9 (CH_2_)	3.44, dd, 13.2, 2.9	3, 17	15
16β		3.68, d, 13.2		
17	10.4 (CH_3_)	0.88, d, 6.8	14, 15, 16	15
18	17.6 (CH_3_)	1.36, s	2, 3, 4	
19α	115.8 (CH_2_)	5.18, br s	6, 7, 8	
19β		5.56, br s		
20	22.8 (CH_3_)	1.39, s	10, 11, 12	
21	175.6 (C) ^c^	-		
22	34.7 (CH_2_)	2.33, m ^b^	21	23
23	25.1 (CH_2_)	1.62, m ^b^	21, 22, 25	22
24	29.0 (CH_2_) ^d^	1.25, m ^b^		
25	28.8 (CH_2_) ^d^	1.30, m ^b^		
26	31.7 (CH_2_)	1.26, m ^b^	24	
27	22.6 (CH_2_)	1.27, m ^b^		28
28	14.1 (CH_3_)	0.86, t, 7.3	26, 27	27

^a^ Chemical shift values are in ppm relative CDCl_3_ residual signals; ^b^* δ* values were obtained by the assistance of the HSQC-edited spectrum; ^c^ The *δ*_C_ values were obtained by means of the HMBC correlations; ^d^ Values can be exchanged.

Comparison of NMR data (^1^H-NMR, ^13^C-NMR) of compounds **1** and **2** revealed several structural similarities and differences. For instance, in compound **2** the nine-membered ring across C-2/C-9 was intact and the spin system formed by protons H-4/H-6 was composed of two oxygen-bearing methines and a methylene, in compound 1 the α,β-unsaturated aldehyde moiety was present instead. The exocyclic methylene (C-19) was attached to C-7 based on the ^2,3^*J* HMBC correlations of H_2_-19 with C-6, C-7 and C-8. This part of the molecule showed very weak or broadened ^13^C and ^1^H-NMR signals, likely due to the existence of a slow equilibrium between different conformations across C-2/C-9, as reported previously for other briarelline analogues [5,6]. On the other hand, the tricyclic structure comprising the tetrahydrofurane ring, the cyclohexanone ring and the seven-membered ether ring across C-3/C-16 was found to be identical in compounds **1** and **2**. The presence of four rings and the structural features shared with compound **1** suggested that compound **2** was a member of the briarellin group. Comparison of NMR data of compound **2** with other briarellins indicated a close structural similarity with briarellin E (**4**) [2], with the presence of a ketone at C-12 being the only difference between compound **2** and briarellin E (**4**). As it was expected, the carbonyl group at C-12 in compound **2** produced a deshielding effect on its adjacent carbons C-11 and C-13, which appeared downfield (*δ*_C_ 75.7, 39.4) in comparison with the same carbons in briarellin E (**4**) (*δ*_C_ 71.6, 24.8). Overall 1D-NMR (^1^H-NMR, ^13^C-NMR, DEPT) and 2D-NMR data (HSQC, COSY, TOCSY and HMBC) (Table 2) confirmed the assignment of the planar structure of compound **2** as briarellin S.

The three-dimensional structure of compound **2** was established by DPFGSE-NOE experiments (Figure 3), which indicated that compounds **1** and **2** have the same relative stereochemistry in all the common chiral centers. Irradiation on H-6 and H-1 produced enhancements on protons H_3_-18 and H-4, respectively. Thus, H-6 was assigned as having α-configuration and H-4 was assigned as having β-configuration. The absolute stereochemistry of natural briarellin E (**4**) was not determined, however Corminboeuf and colleagues carried out the enantioselective total synthesis of **4** [9]. Given that briarellins E (**4**) and S (**2**) have the same chiral centers, they likely have the same absolute configuration.

**Figure 4:**
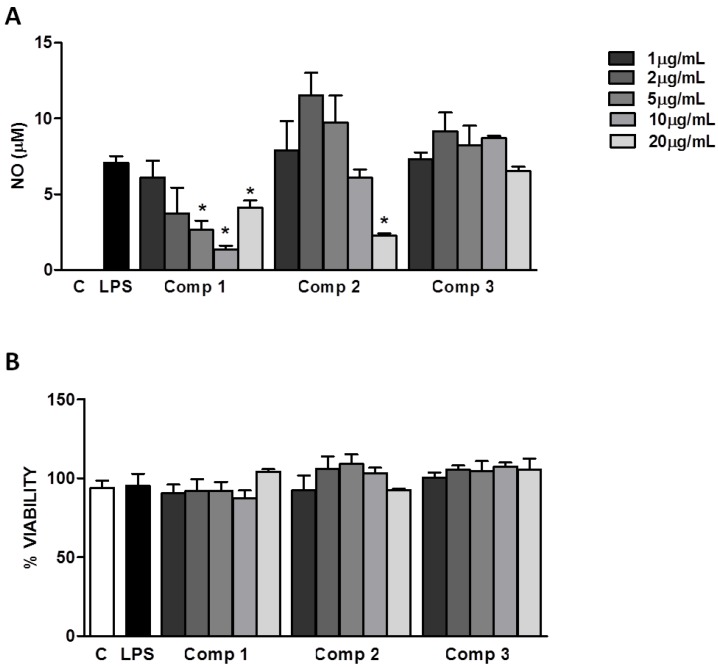
Inhibition of nitric oxide (NO) production induced by lipopolisacharides (LPS) in macrophages. (**A**) Peritoneal macrophages from C57Bl/6 mice were treated with different concentrations of compounds **1**–**3** (1, 2, 5, 10, or 20 μg/mL) 1 h before the stimulus with LPS (1 μg/mL). After 24 h of stimulus with LPS, supernatants were collected for NO determination. (**B**) Cell viabilities were assessed using 3-(4,5-dimethylthiazol-2-yl)-2,5-diphenyltetrazolium bromide (MTT) assay after collection of the supernatant. Results represent mean ± S.E.M. and are representative of two different experiments. * *P* < 0.005 compared with LPS stimulus alone. C, control; Comp, compound.

The anti-inflammatory properties of diterpenes from natural sources have been described previously [10]. However, anti-inflammatory activity of the briarellins family has not been evaluated before. The inhibition of NO production is frequently used as an indicator of anti-inflammatory activity. NO is produced by a family of enzymes called NO synthases (NOS) and it mediates many physiological processes [11]. Inflammatory conditions lead to production of high levels of NO regulated by the inducible NOS (iNOS) enzyme. NO is produced by many cell types *in vitro* in response to several stimuli such as microbial products (e.g., bacterial lipopolisacharide (LPS)), cytokines, viral proteins, among others [11]. We evaluated the production of NO by primary murine macrophages stimulated with LPS (1 μg/mL) in the presence or absence of different concentrations of compounds **1**–**3**. As it is shown in Figure 4A, compounds **1 **and **2 **inhibited the production of NO with IC_50_’s of 1.71 μg/mL (4.7 μM) and 10.04 μg/mL (20.4 μM), respectively. The levels of NO found at higher concentrations (20 μg/mL) of compound **1** might be attributed to the non pyrogen-free conditions of the isolation and purification process of compounds. Possible contaminations with potential microbial products in the compounds preparations disguise the inhibitory effect expected at these concentrations. Compound **3** did not show any significant effect on the production of NO induced by LPS in macrophages (Figure 4A). Further studies are necessary to find out if compound **3** is able to inhibit other signaling pathways leading to the production of inflammatory mediators different than NO. The inhibition of NO production induced by compounds **1** and **2** is not due to their cytotoxicity, since the inhibitory concentrations (5–20 μg/mL) do not interfere with the cell viability, as it was determined by the 3-(4,5-dimethylthiazol-2-yl)-2,5-diphenyltetrazolium bromide (MTT) method (Figure 4B). Our results indicate that compounds **1** and **2** can control NO production and could be promising anti-inflammatory agents. Other studies should be performed to elucidate the mechanism by which these compounds inhibit the production of NO and if there are other pathways being regulated by them.

## 3. Experimental Section

### 3.1. General Experimental Procedures

Optical rotations were measured on a JASCO P-2000 polarimeter, whereas IR data was obtained using a Shimadzu IRAffinity-1 Fourier transform infrared spectrophotometer. ^1^H, ^13^C, and 2D NMR spectra were collected at a ^1^H resonance frequency on either a Jeol Eclipse+ 400 MHz or Bruker Avance III DRX600 (equipped with a 1.7 mm TCI cryoprobe). Chemical shifts were calibrated internally to the residual signal of the solvent in which the sample was dissolved (CDCl_3_, *δ*_H_ 7.26, *δ*_C_ 77.0). High-resolution mass spectra were obtained on a ThermoFinnigan MAT900XL mass spectrometer. HPLC was carried out using an Agilent 1200 HPLC system equipped with a quaternary pump, a diode array detector and a normal phase silica gel column (Phenomenex Sphereclone, 4.6 mm × 100 mm, 5 μm) at a flow rate of 1 mL/min. Flash chromatographic separations were performed using silica gel type H (10–40 μm, Aldrich) and silica gel 60 (40–63 μm, EMD), respectively. Merck TLC sheets (silica gel 60 F254) were used for analytical TLC (aluminum-supported, layer-thickness 200 µm).

### 3.2. Animal Material

The octocoral *Briareum asbestinum *(Order Alcyonacea, Family Briaridae) was collected by hand using SCUBA at 10 m in Bastimentos National Park, located in the Caribbean off the coast of Bocas del Toro, Panama in November 2009. The coral specimen was identified as *Briareum asbestinum *(Pallas) based on its morphology and SEM-micrographs of the coral sclerites in the Smithsonian Tropical Research Institute. A reference specimen is deposited at INDICASAT’s CDDB under the number GLBO-231109-01.

### 3.3. Extraction and Isolation

The organism (1265 g) was minced and exhaustively extracted with CH_2_Cl_2_ and MeOH. The organic extract was evaporated *in vacuo* to give a dark oily residue (49.6 g). The CH_2_Cl_2_-MeOH extract (21.7 g) was chromatographed by column chromatography on silica gel eluted with a stepwise gradient of 0%–100% EtOAc in hexanes followed by 0%–100% MeOH in EtOAc to yield 10 fractions (A–J). Fraction E (82.7 mg) was purified by HPLC (reverse phase Synergy-Fusion column eluted with a gradient of 60%–100% MeCN in water in 60 min at 1 mL/min) to yield 15 fractions. Fraction 10 yielded 10 mg of pure *seco*-asbestinin (**3**) [12]. Fraction F was concentrated (266.2 mg) and further chromatographed on silica gel eluted with a stepwise gradient of CHCl_3_-*i*PrOH (150:1, 100:1, 50:1) followed by CHCl_3_-EtOH (25:1, 10:1, 1:1) to yield 22 fractions (1–22). Fraction 13 (33 mg) was purified by HPLC (5 μm Silica gel Sphereclone column eluted with a gradient of 40%–100% EtOAc in hexanes in 130 min at 1.0 mL/min) to yield 20 subfractions, denoted I–XX. Subfraction VIII contained 6.8 mg of pure briarellin S (**2**), and subfraction XVIII contained 0.7 mg of pure *seco*-briarellinone (**1**). 

*seco*-Briarellinone(**1**): Colorless oil; [α]^20^_D_ +38.1 (*c* 0.4, CHCl_3_); IR (film) ν_max_ 3406, 2916, 2848, 1722, 1581, 1462, 1377, 1242, 1111, 1076 cm^−1^; ^1^H and ^13^C NMR see Table 1; HRESI-TOF-MS *m/z* [M + Na]^+^ 387.1775 (calcd for C_20_H_28_O_6_Na, 387.1778). 

Briarellin S (**2**): Colorless oil; [α]^20^_D_ +36.1 (*c* 4.1, CHCl_3_); IR (film) ν_max_ 3452, 2929, 2873, 1720, 1456, 1377, 1247, 1166, 1112, 1080, 1006 cm^−1^; ^1^H and ^13^C NMR see Table 2; HRESI-TOF-MS *m/z* [M + Na]^+^ 515.2975 (calcd for C_28_H_44_O_7_Na, 515.2979).

### 3.4. Cell Culture and NO Determination

To determine the anti-inflammatory capacity of compounds **1–3**, thioglycolate elicited macrophages from C57Bl/6 mice were used. Five days after i.p. instillation of 2 mL of thioglycolate 3%, peritoneal macrophages were obtained by washing the cavity with chilled RPMI. Cells were seeded in RPMI with 10% FCS at 2 × 10^5^/well in 96-well plates. Cells were stimulated with LPS (1 μg/mL) in the presence or absence of different concentrations of compounds **1–3** as described in the legend of Figure 4. Supernatants were collected 24 h after stimuli and were stored at −20 °C until its use. Negative (without stimulus) and positive (LPS stimulus alone) controls were performed in the presence of dimethyl sulfoxide (DMSO) since the compounds were solubilized in DMSO. The concentration of NO was measured using the Griess Reagent System from Promega. All the measurements were performed in triplicate following the manufacturer’s instructions.

### 3.5. Cytotoxicity Testing

To evaluate the cytotoxicity of compounds **1–3**, we used the MTT (Methylthiazolyldiphenyl-tetrazolium bromide) assay. Cell supernatants were removed for NO determination and were added to each well, 100 μL of MTT at a concentration of 0.5 mg/mL dissolved in RPMI. The assay plate was incubated overnight at 37 °C in 5% of CO_2_ atmosphere. In this assay the MTT is reduced to formazan by the activity of NAD-dependent dehydrogenase of living cell mitochondria to form a purple product. The supernatants were removed and formazan crystals were dissolved in 100 µL of 0.04 M HCl in *i*PrOH. The color was analyzed at 570 nm using a micro-ELISA plate reader. The percent of viable cells was calculated using the formula: % viability = (OD_sample_ × 100%)/(OD_control_). Cells non stimulated and cultured in medium plus FCS represented 100% of viability.

### 3.6. Statistical Analysis

Data are presented as mean ± S.E.M. Results were analyzed using a statistical software package (GraphPad Prism 5). Statistical analyses were performed by unpaired *t* test. A significant difference between groups was considered if *p* < 0.05. Inhibitory concentration 50% (IC_50_) values were calculated adjusting a sigmoidal dose-response curve following GraphPad Prism 5 procedure.

## 4. Conclusions

This is the first report on natural products from the octocoral *Briareum asbestinum* collected in Panama. As it is shown here, *B. asbestinum* continues to be a good producer of novel diterpenes with complex chemical structures. The compounds reported here are the first members of briarellins and *seco*-briarellins families with a ketone group at position C-12. Additionally, this work describes anti-inflammatory activity not reported before for the briarellins and *seco*-briarellins groups. 

## Supplementary Files

Supplementary File 1: Supplementary Information (PDF, 2942 KB)
