# Supplementary material for: seco-Briarellinone and Briarellin S, Two New Eunicellin-Based Diterpenoids from the Panamanian Octocoral Briareum asbestinum
Source: Mar Drugs. 2012 Nov 21;10(11):2608–17. doi: 10.3390/md10112608 (PMC3509539; doi:10.3390/md10112608)

Supplementary Information

Figure S1. *seco*-Briarellinone, ^{1}H NMR spectrum.

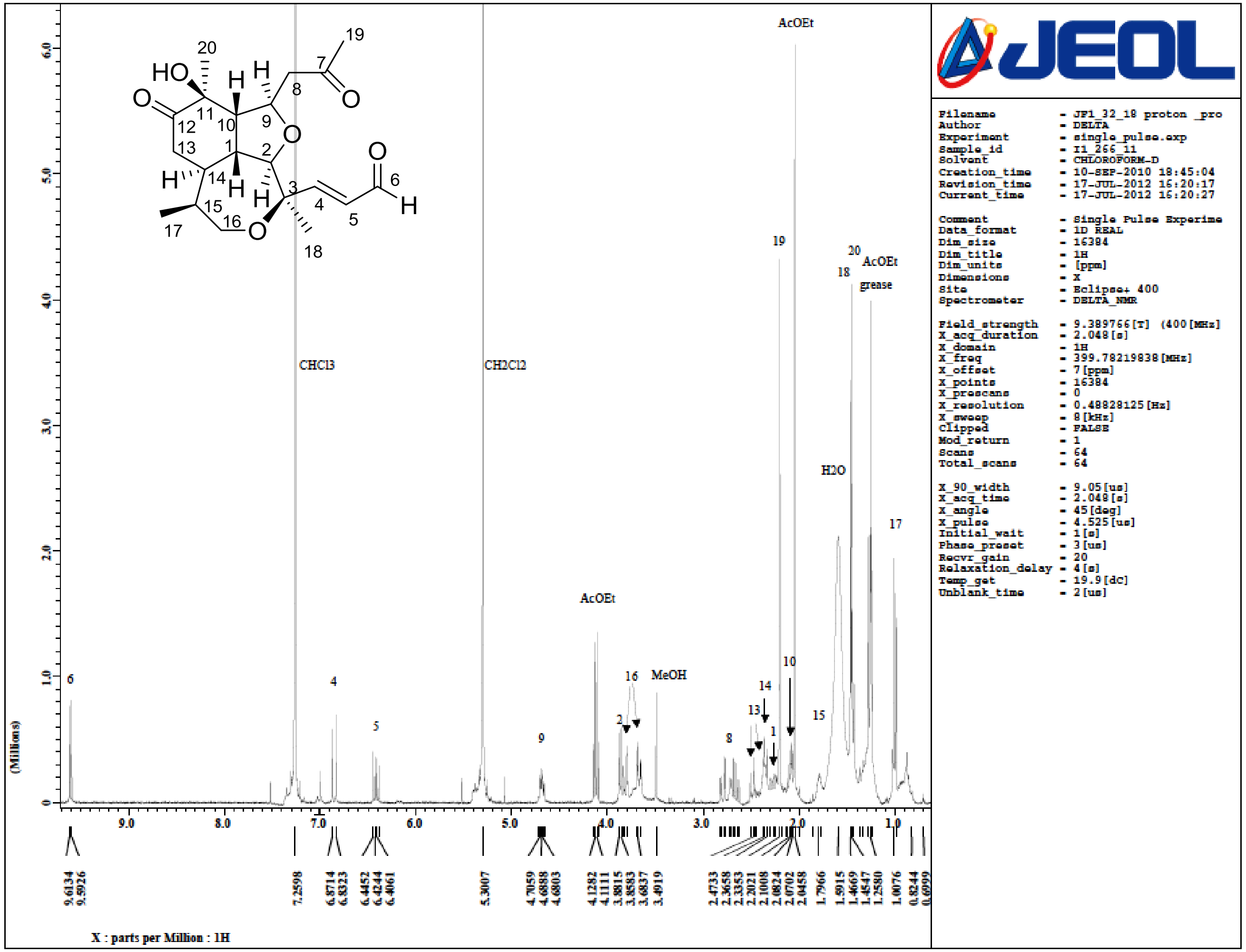

Figure S2. *seco*-Briarellinone, 13C NMR spectrum. The inset shows the total view of spectrum.

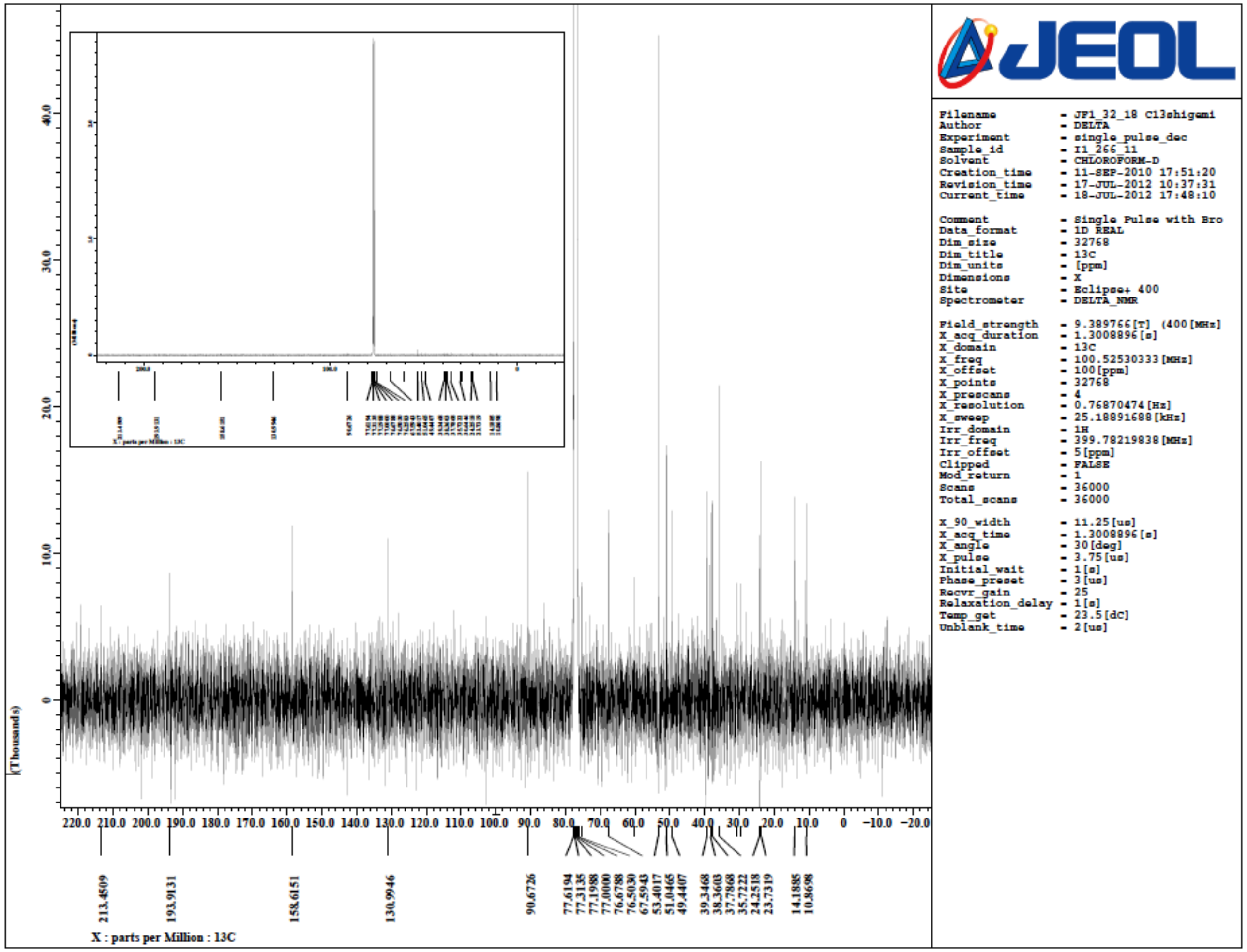

Figure S3. *seco*-Briarellinone, expanded view of the 13C NMR spectrum (9–80 ppm).

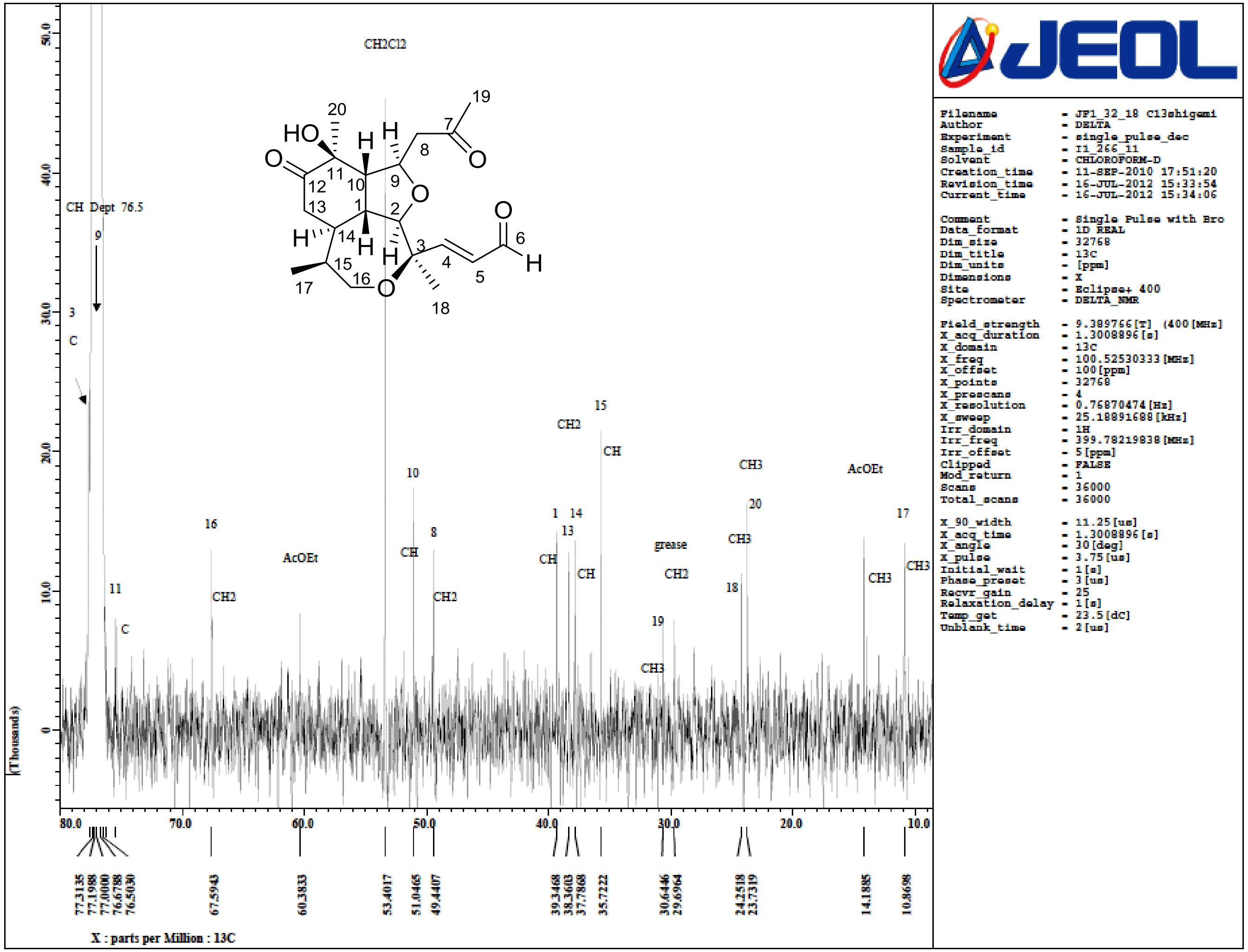

Figure S4. *seco*-Briarellinone, expanded view of the 13C NMR spectrum (80–225 ppm).

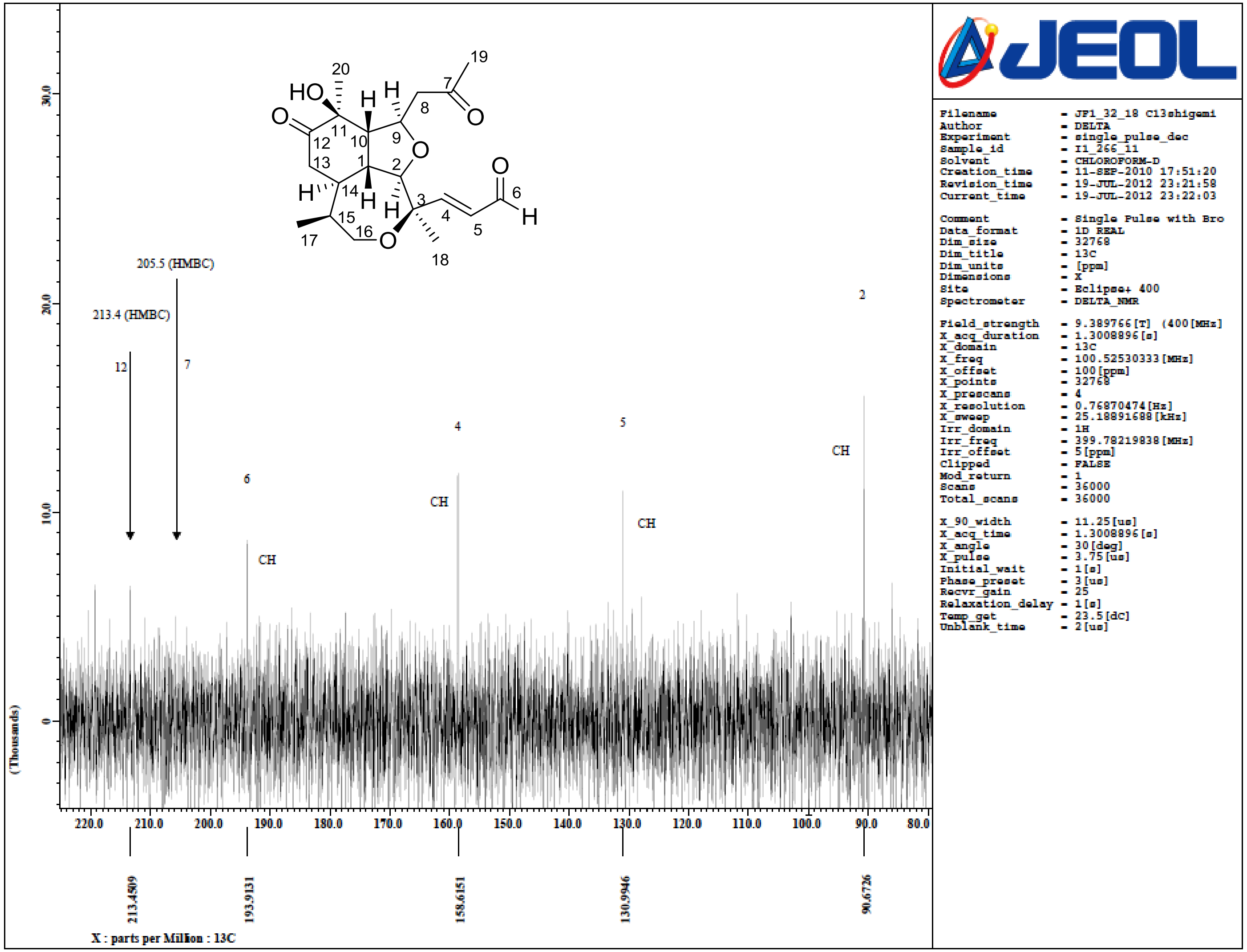

Figure S5. *seco*-Briarellinone, COSY spectrum.

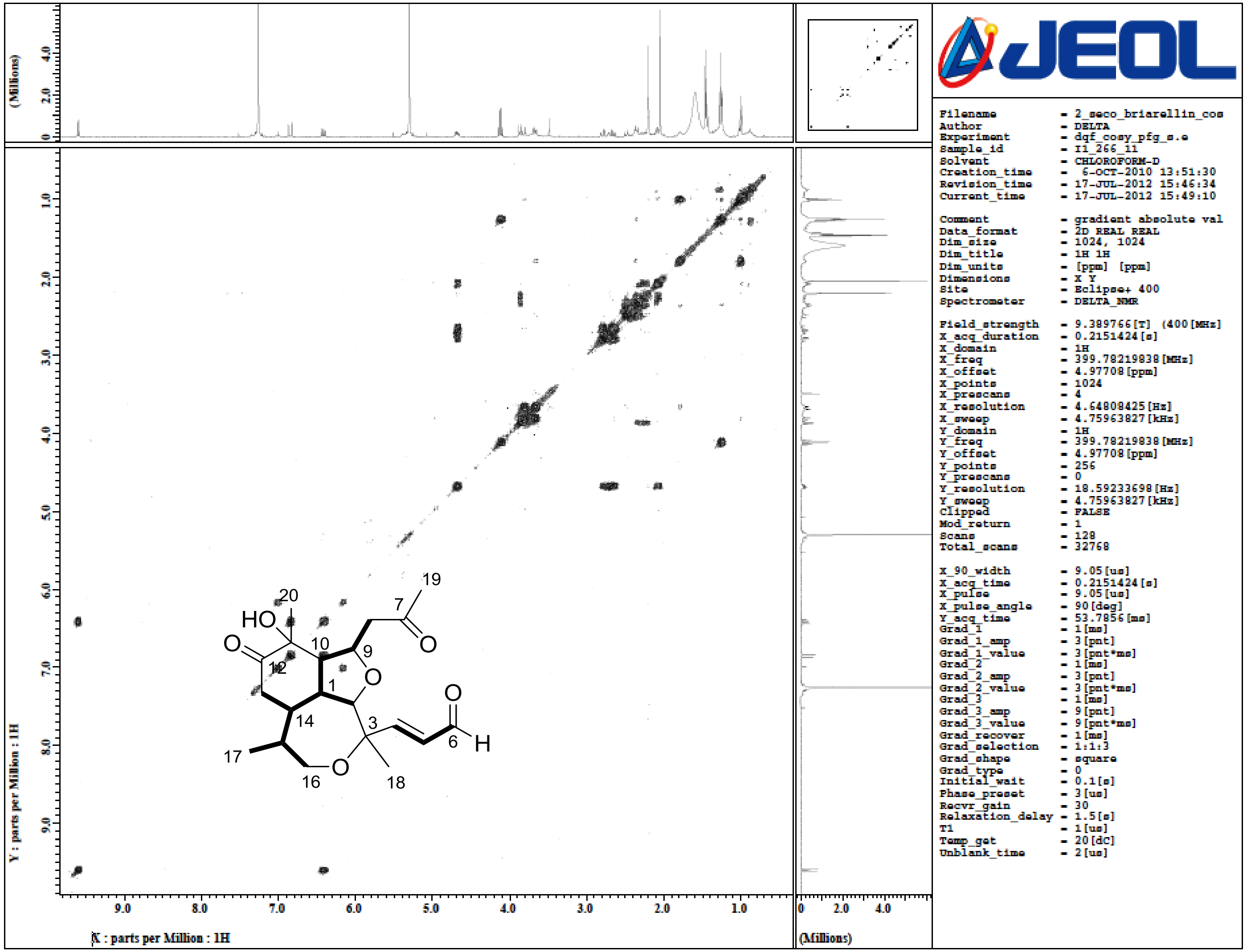

Figure S6. *seco*-Briarellinone, HSQC spectrum.

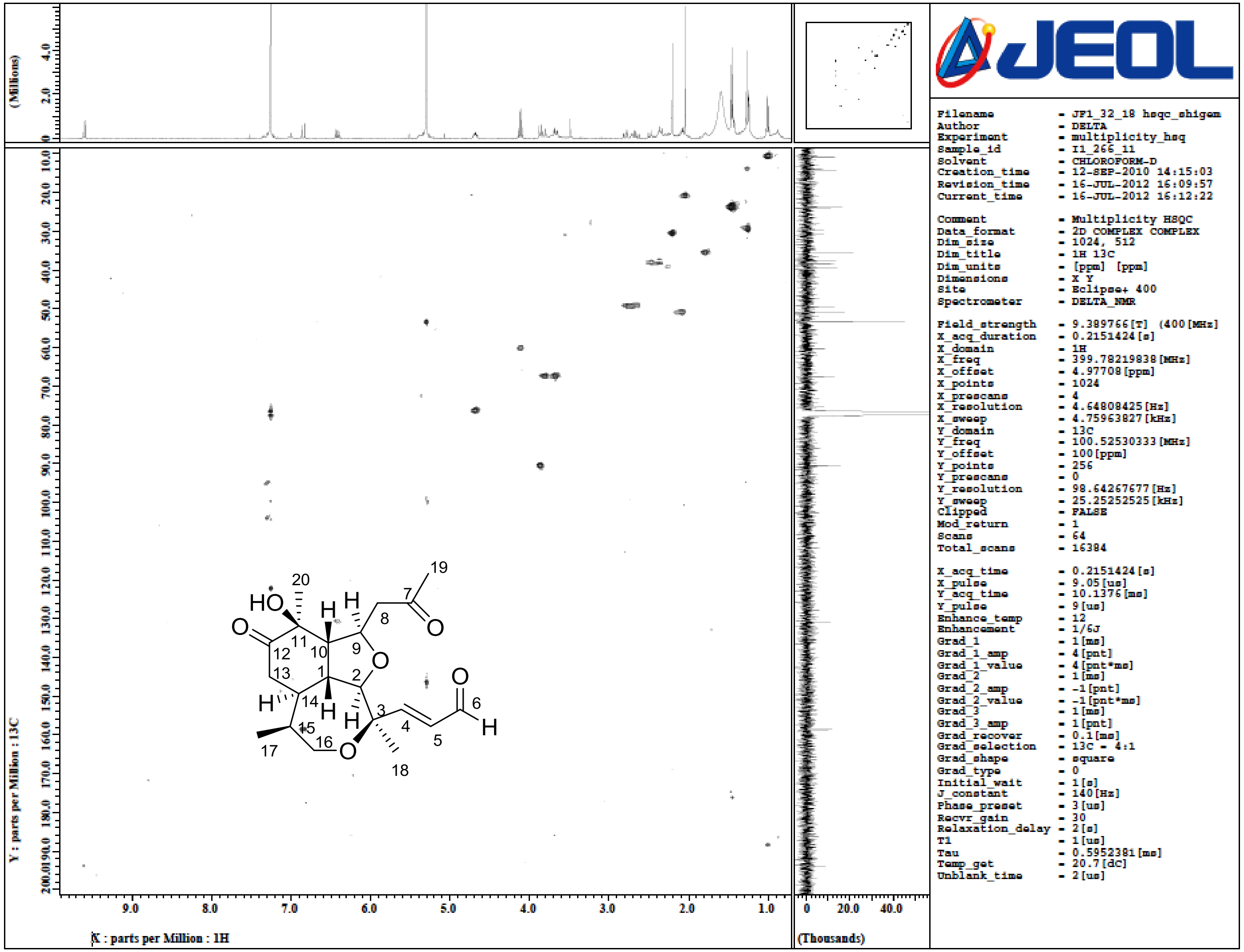

Figure S7. *seco*-Briarellinone, HMBC spectrum.

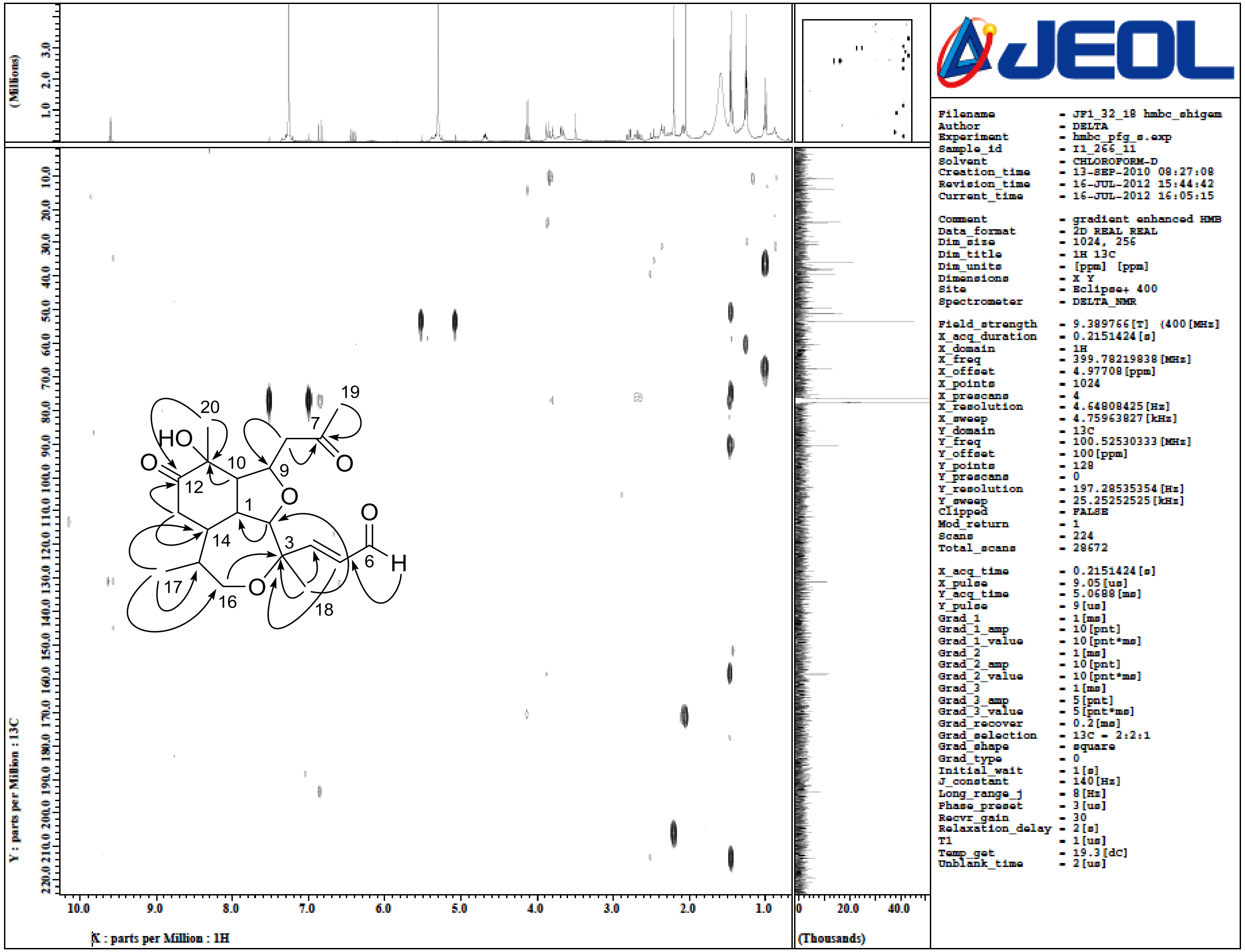

Figure S8. *seco*-Briarellinone, selective DPFGSE-NOE spectrum by irradiation of H-1.

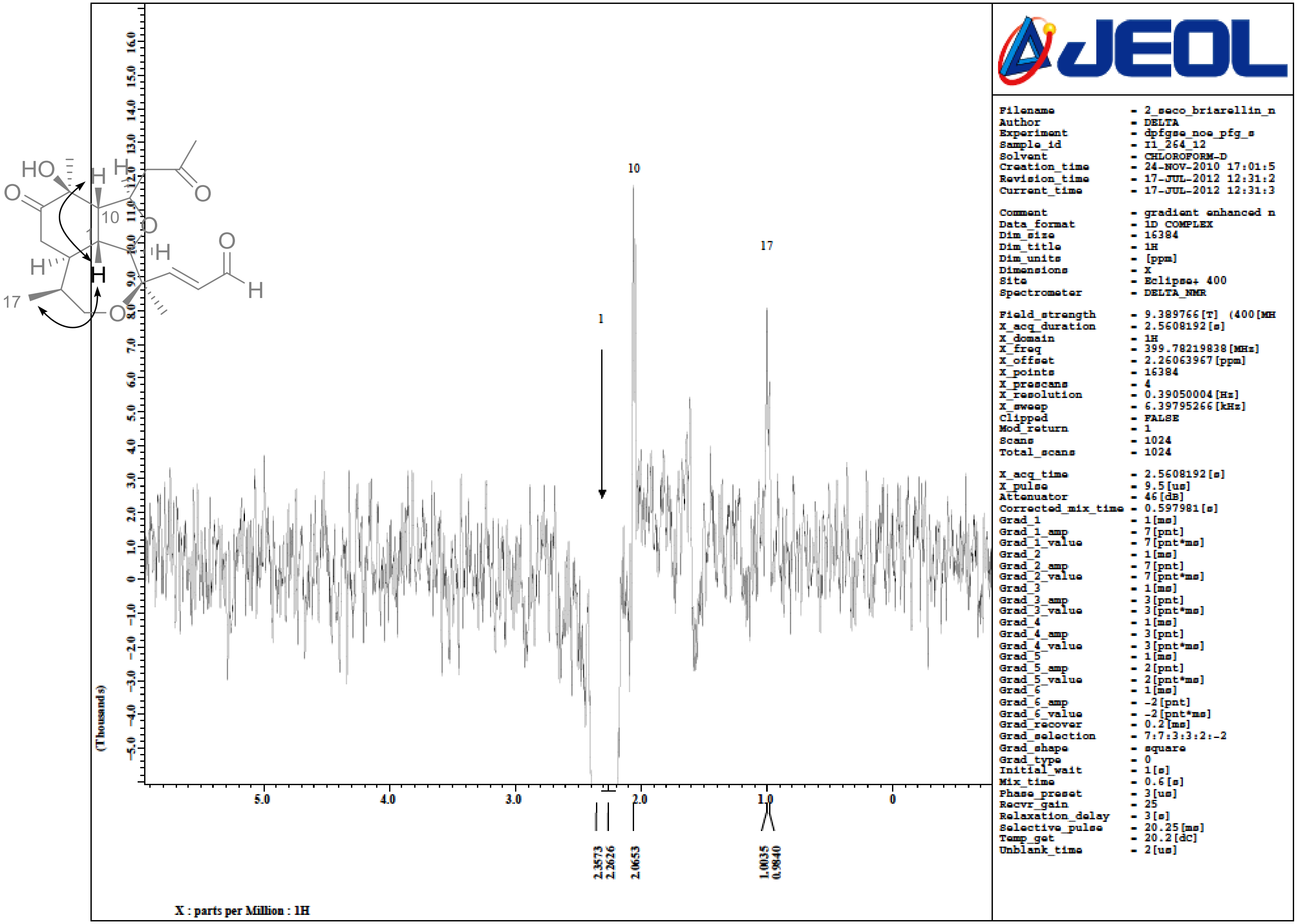

Figure S9. *seco*-Briarellinone, selective DPFGSE-NOE spectrum by irradiation of H-2.

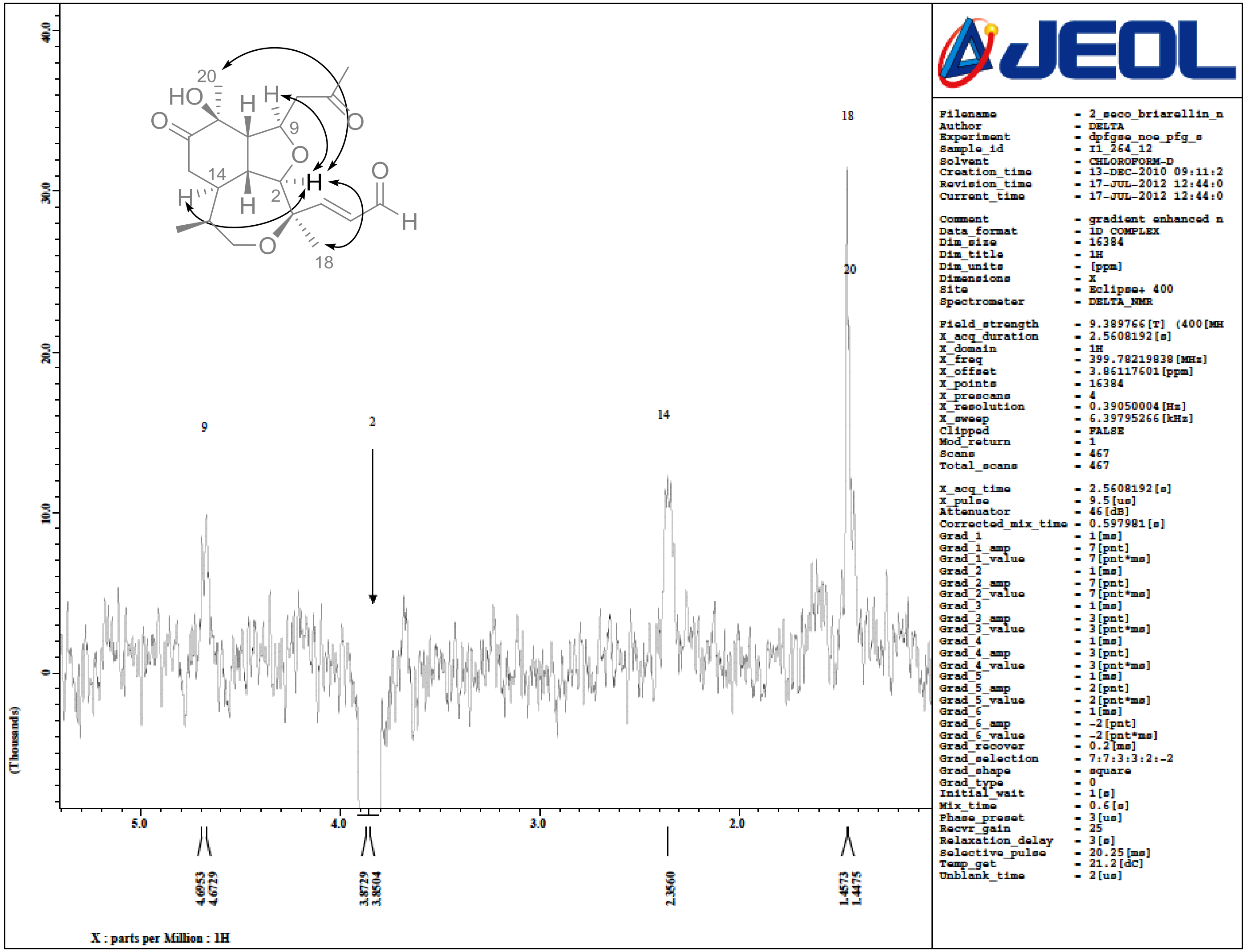

Figure S10. *seco*-Briarellinone, selective DPFGSE-NOE spectrum by irradiation of H-9.

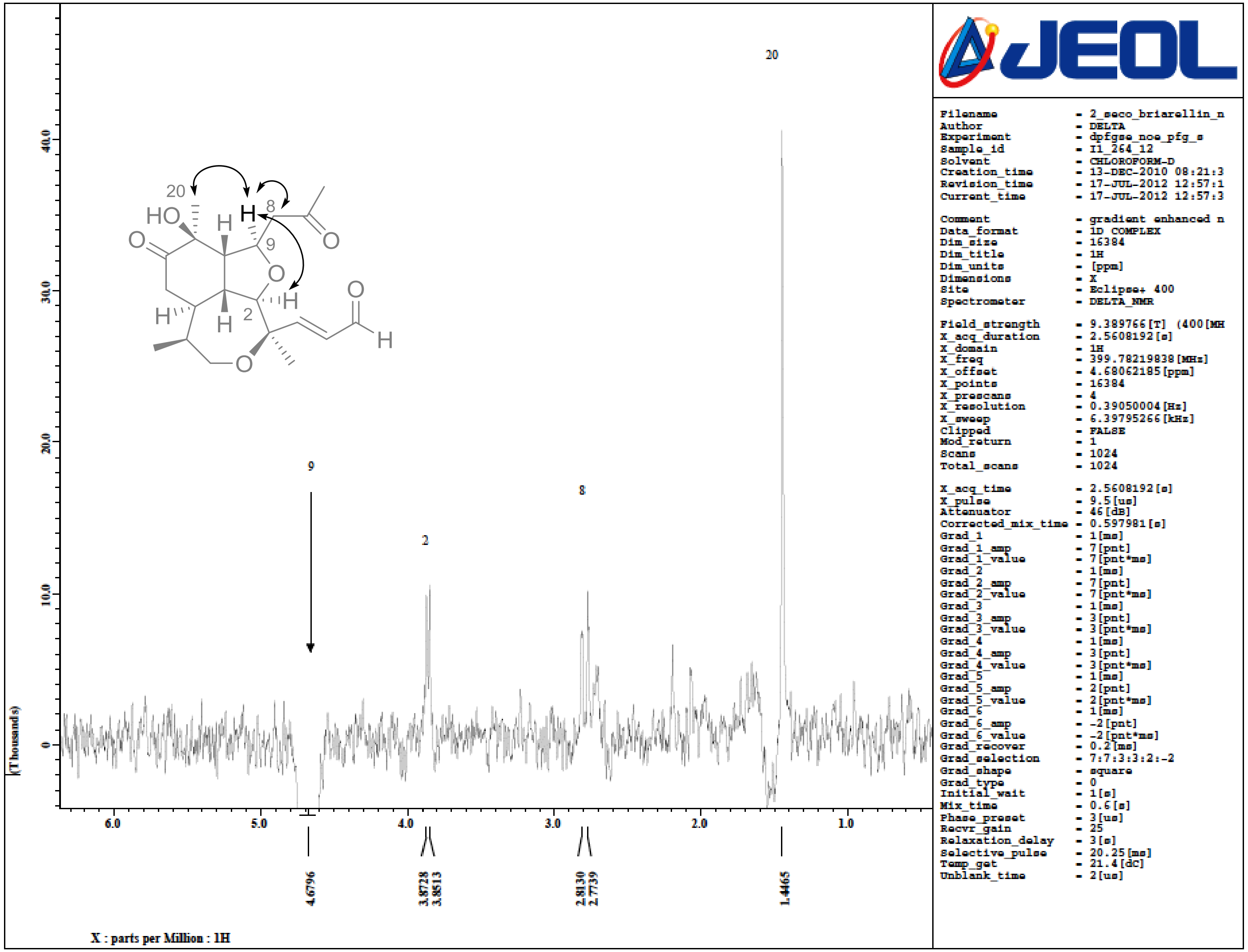

Figure S11. *seco*-Briarellinone, selective DPFGSE-NOE spectrum by irradiation of H-15.

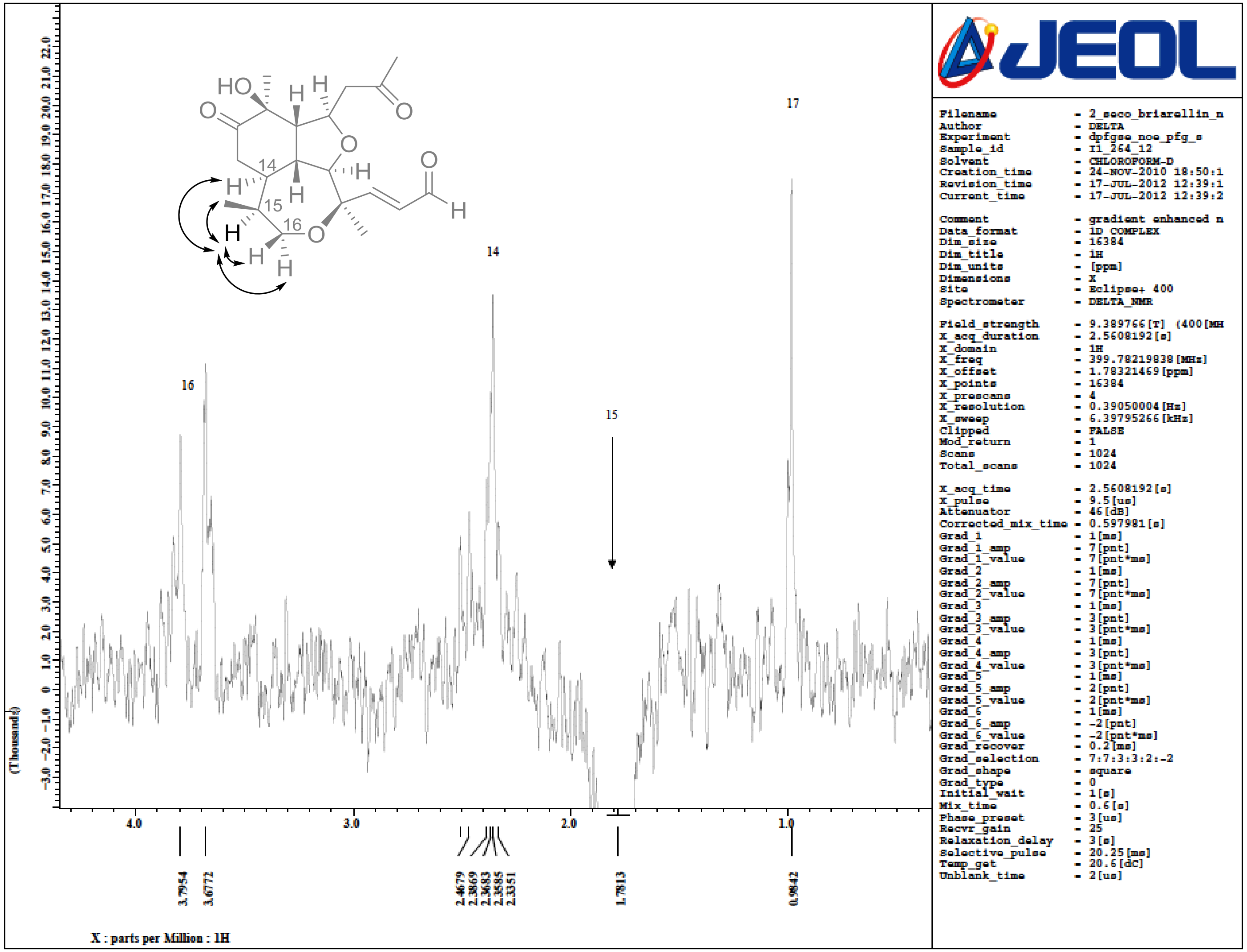

Figure S12. *seco*-Briarellinone, NOESY spectrum.

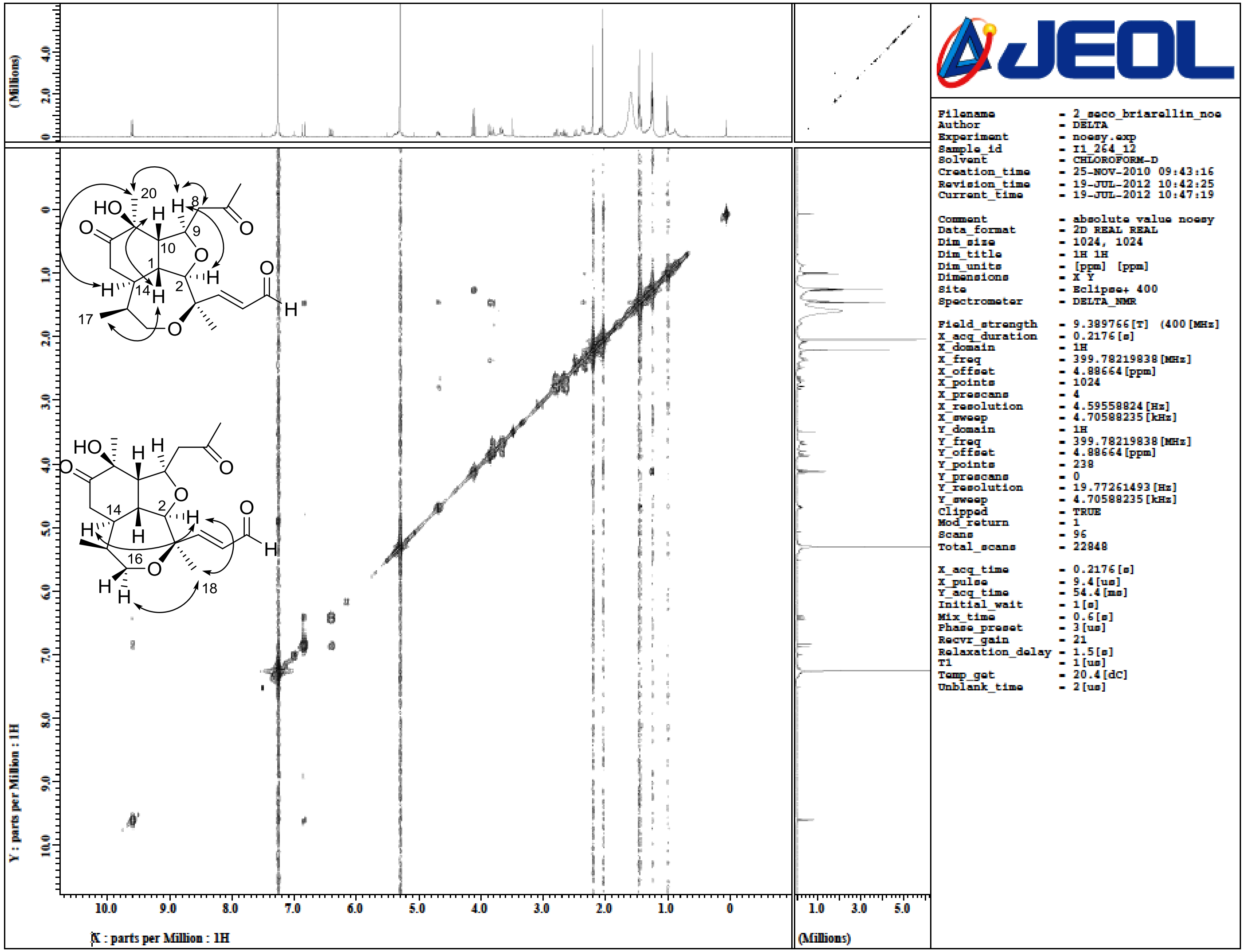

Figure S13. *seco*-Briarellinone, HRMS-ESI-TOF spectrum.

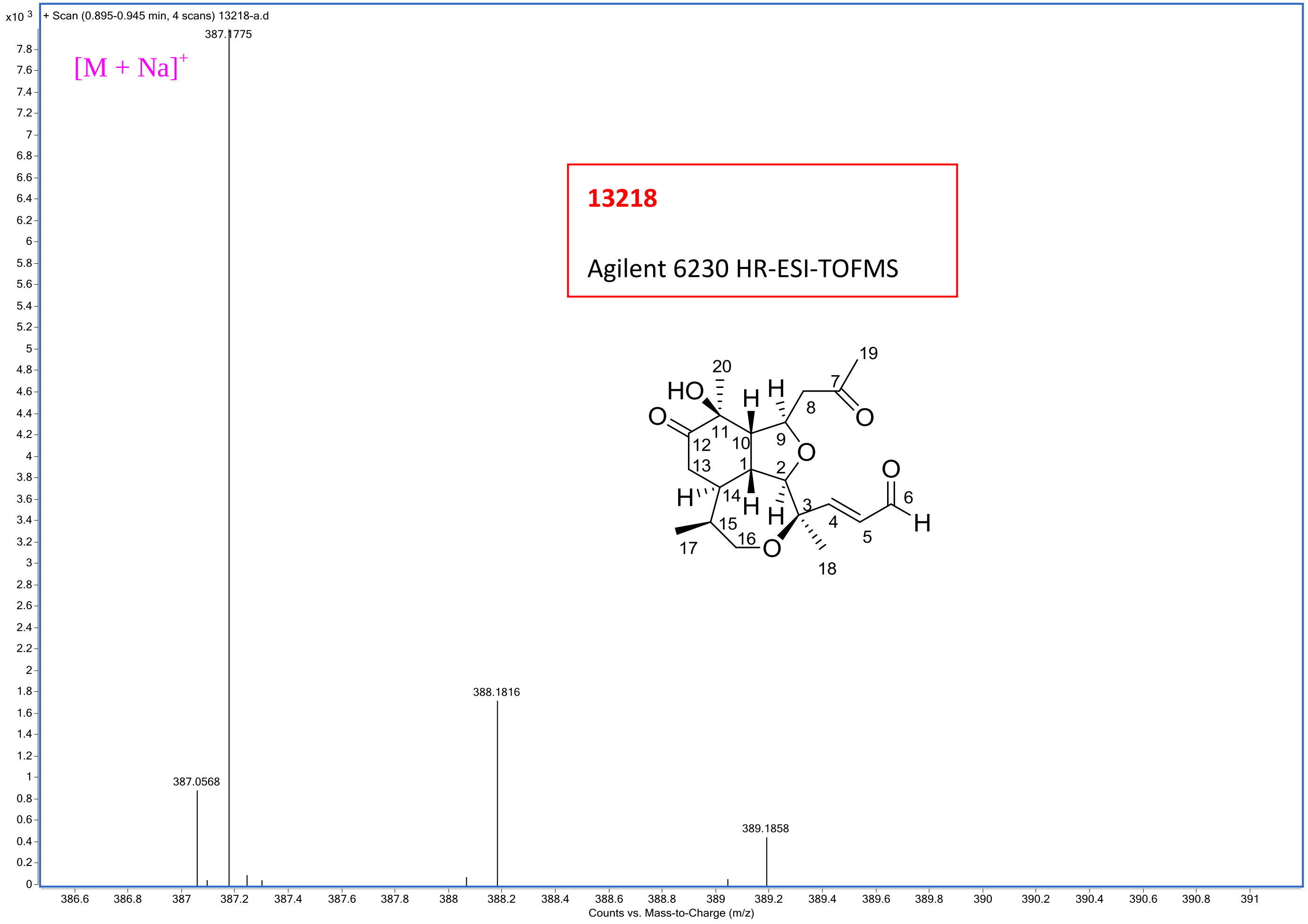

Figure S14. Briarellin S, 1H NMR spectrum.

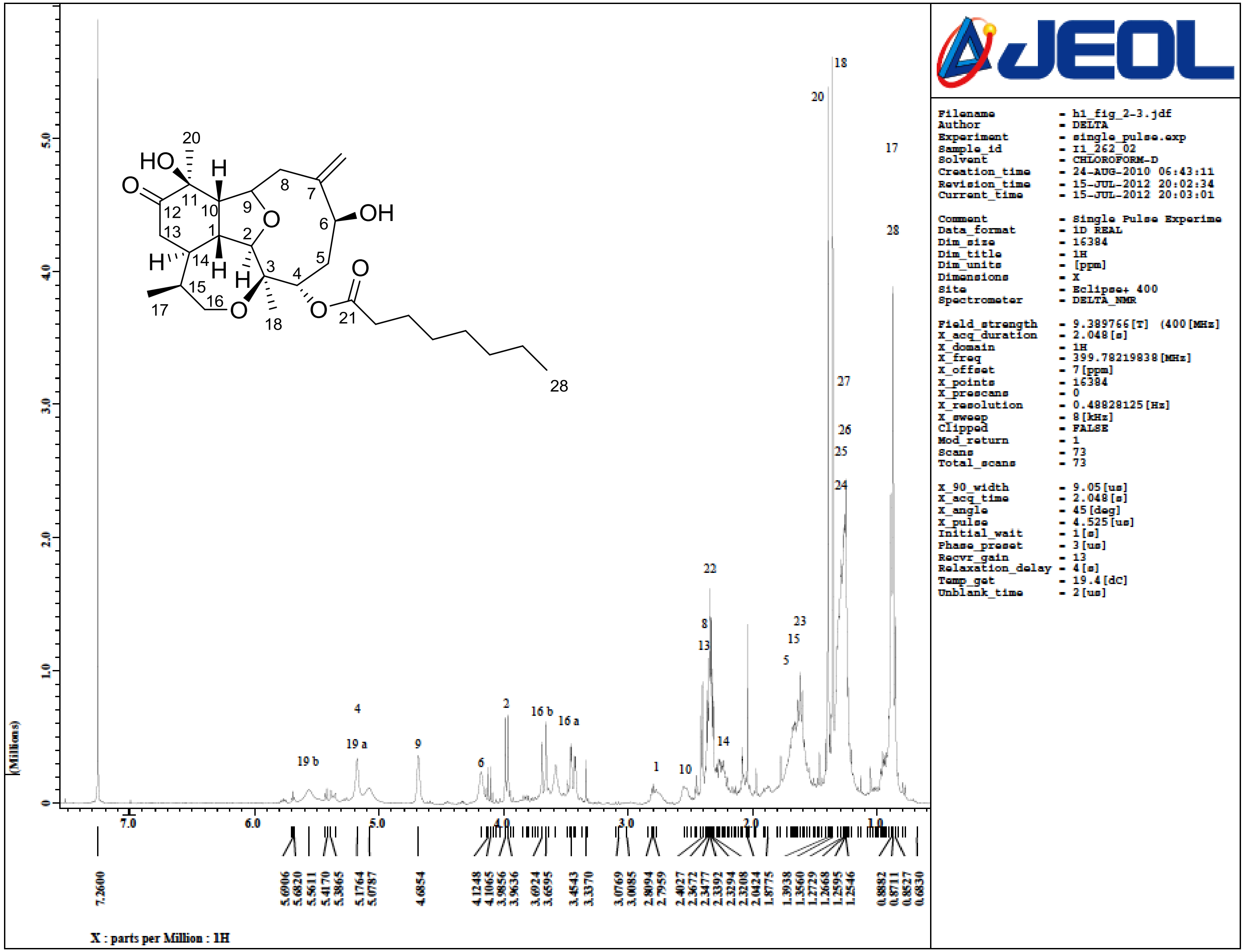

Figure S15. Briarellin S, 13C NMR spectrum.

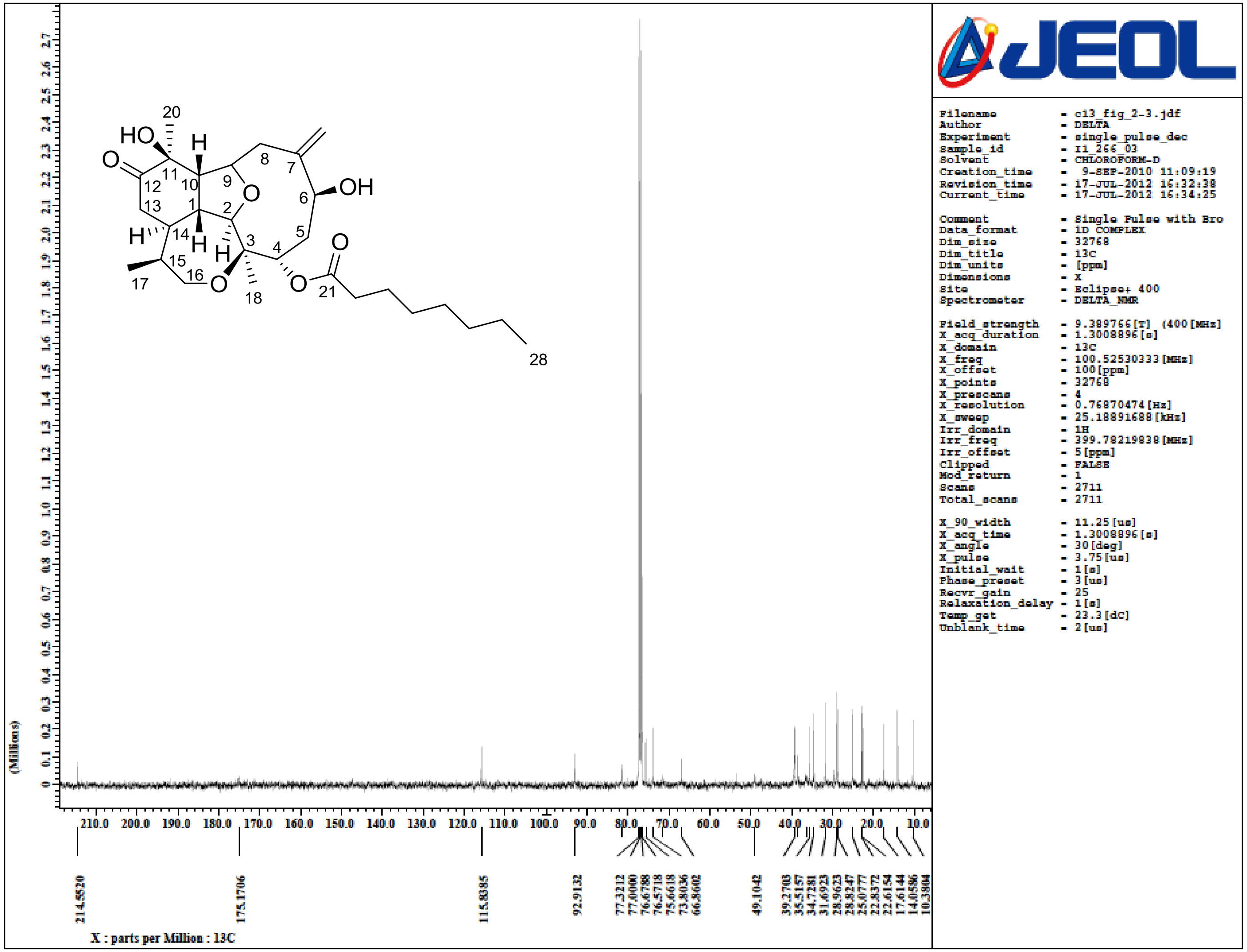

Figure S16. Briarellin S, expanded view of the 13C NMR spectrum (10–40 ppm).

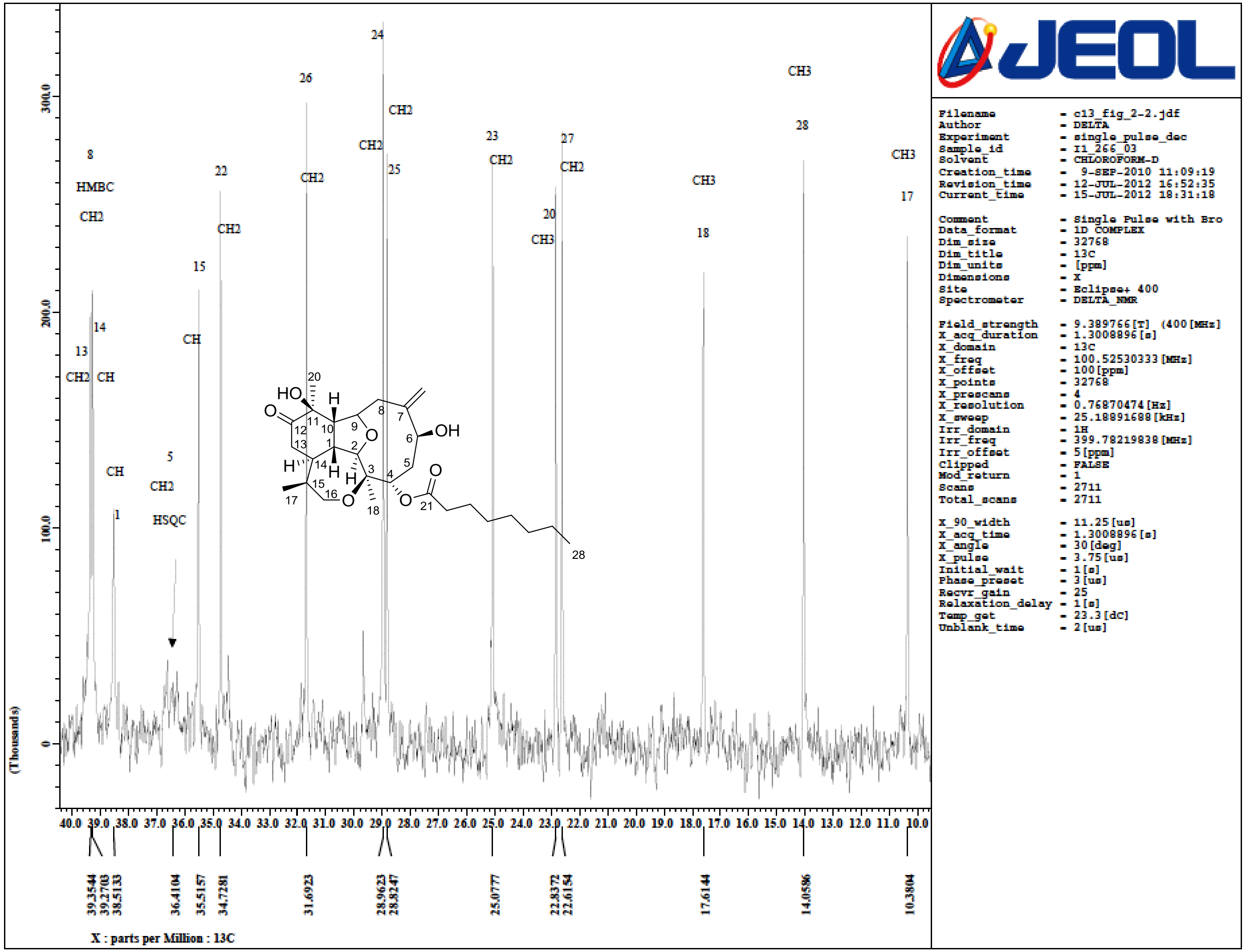

Figure S17. Briarellin S, expanded view of the 13C NMR spectrum (40–80 ppm).

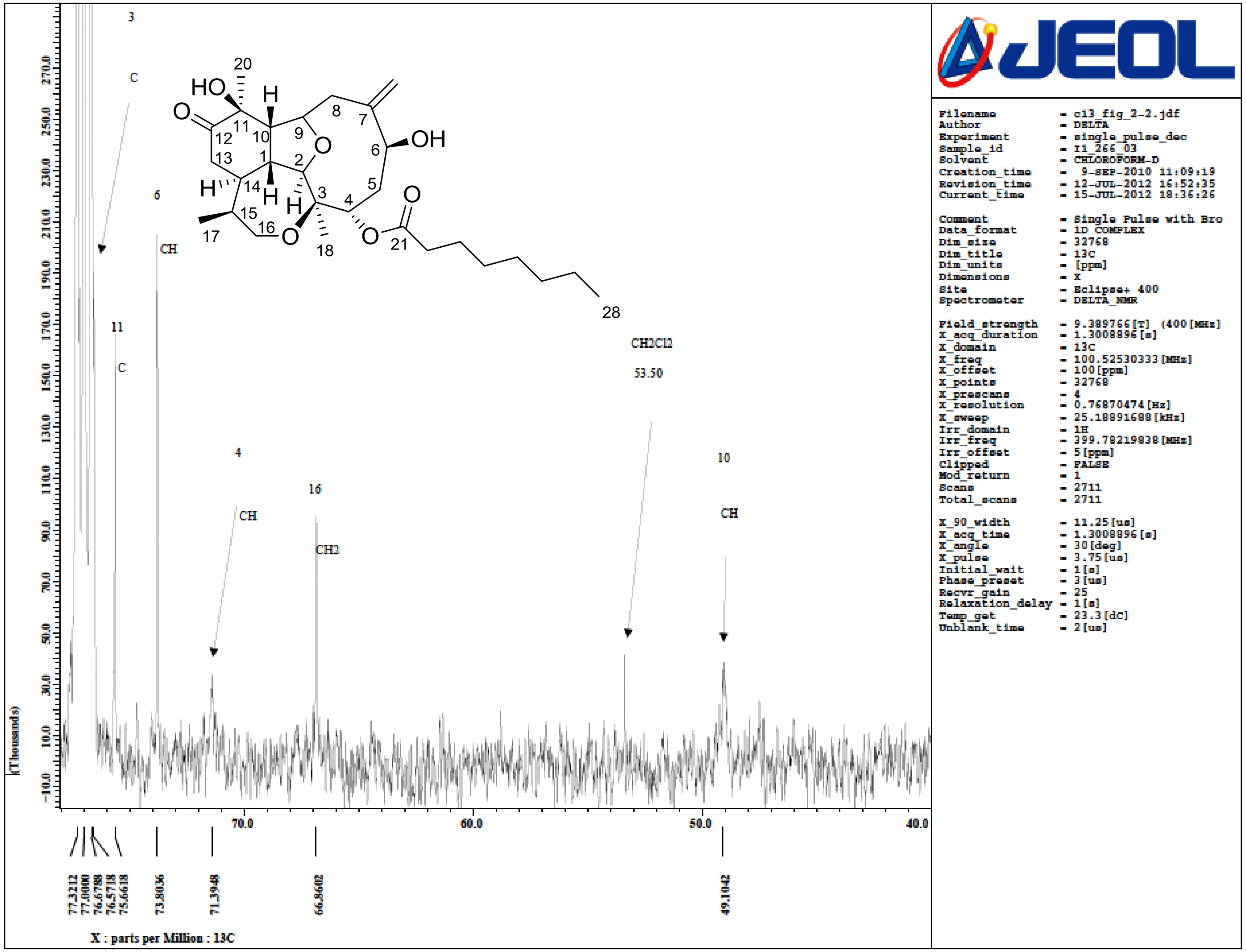

Figure S18. Briarellin S, expanded view of the 13C NMR spectrum (78–220 ppm).

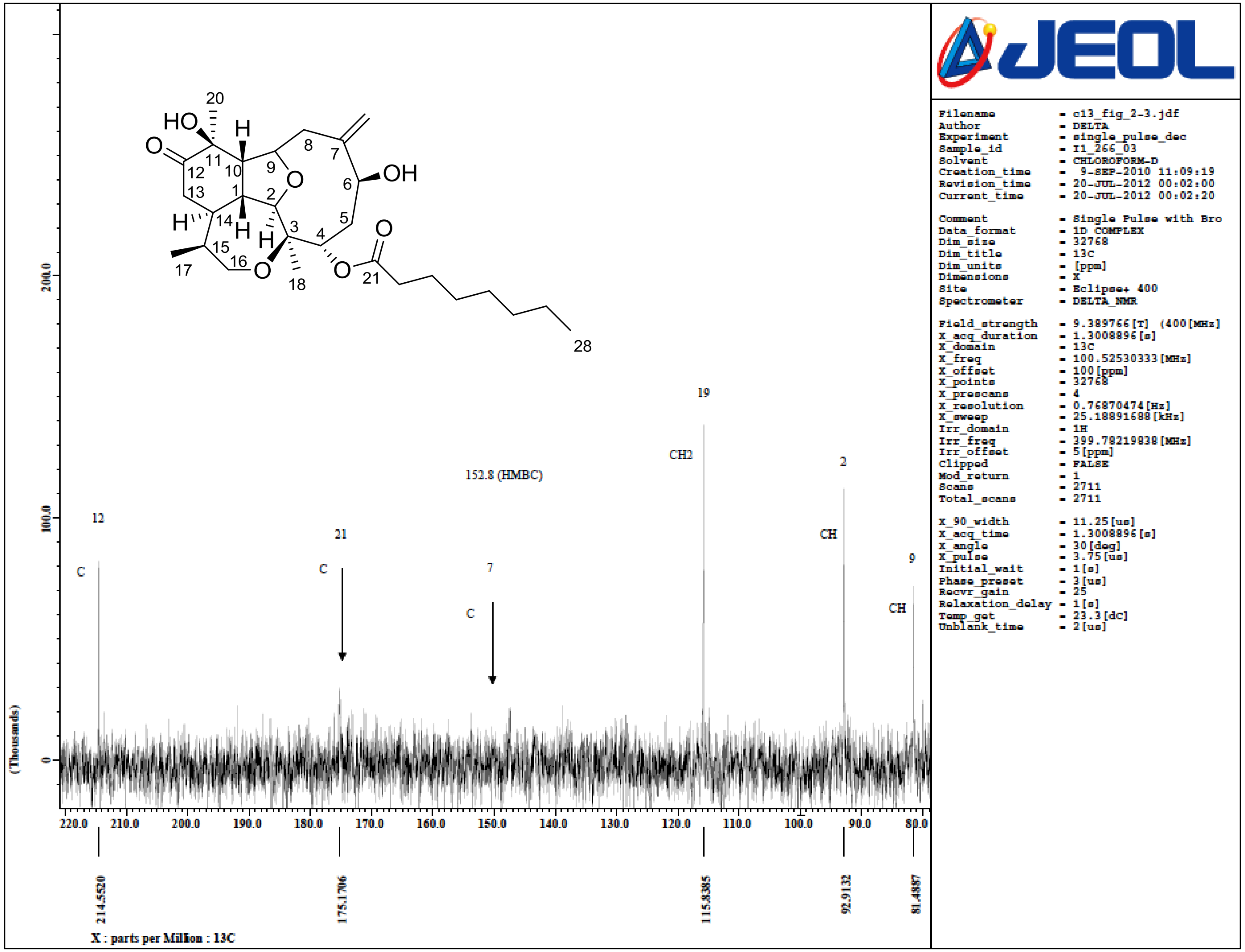

Figure S19. Briarellin S, COSY spectrum.

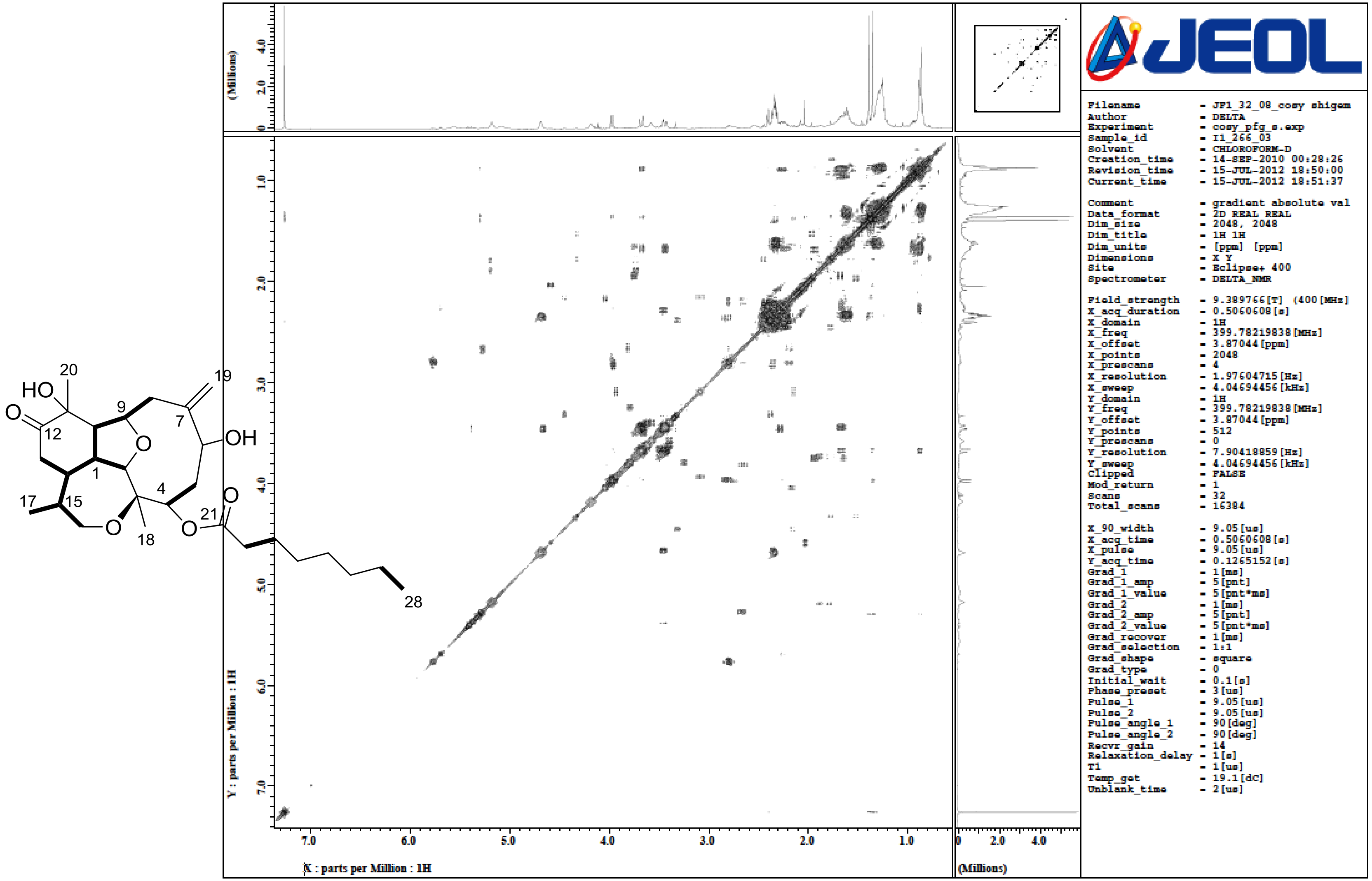

Figure S20. Briarellin S, TOCSY spectrum.

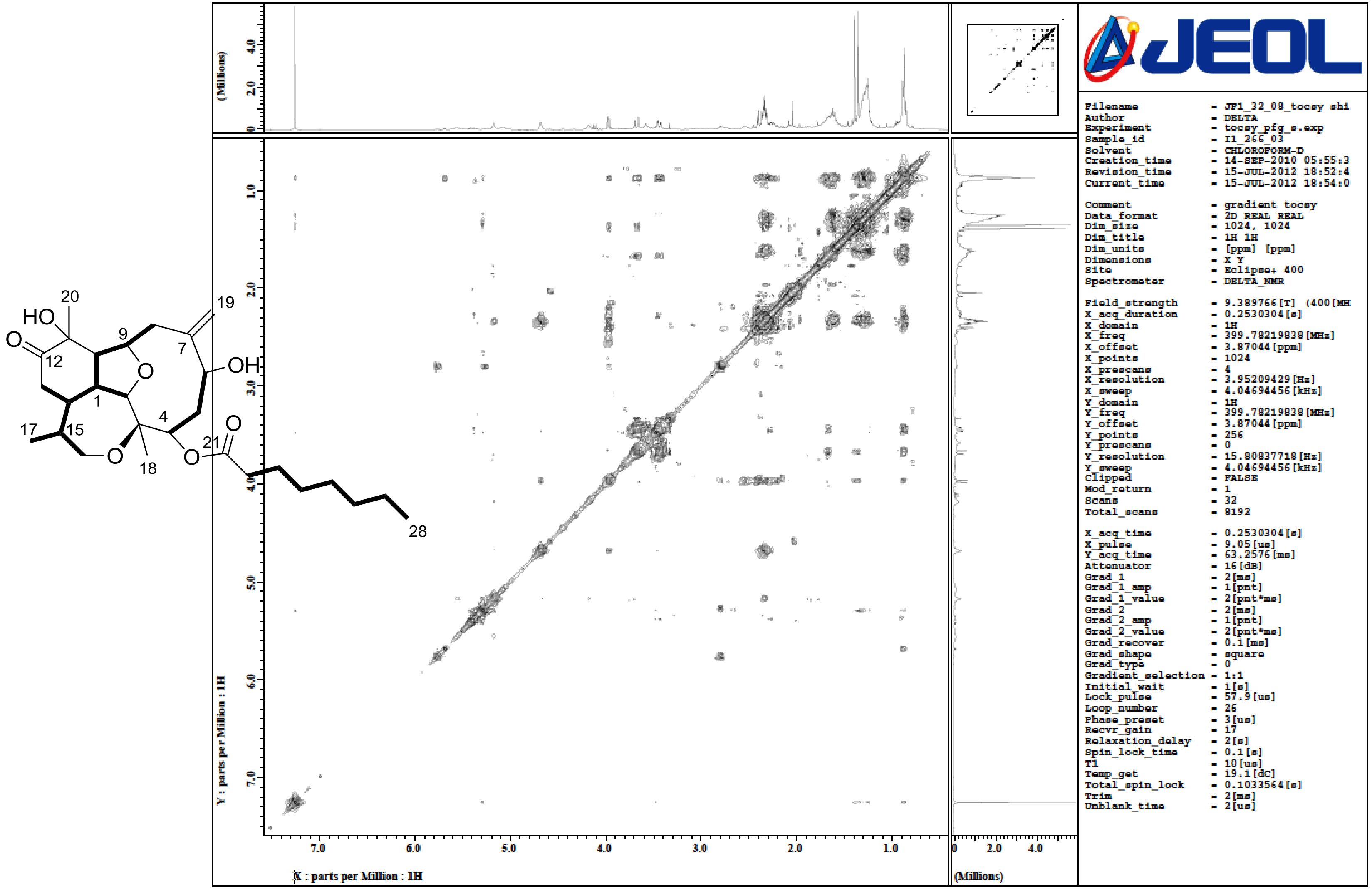

Figure S21. Briarellin S, HSQC spectrum.

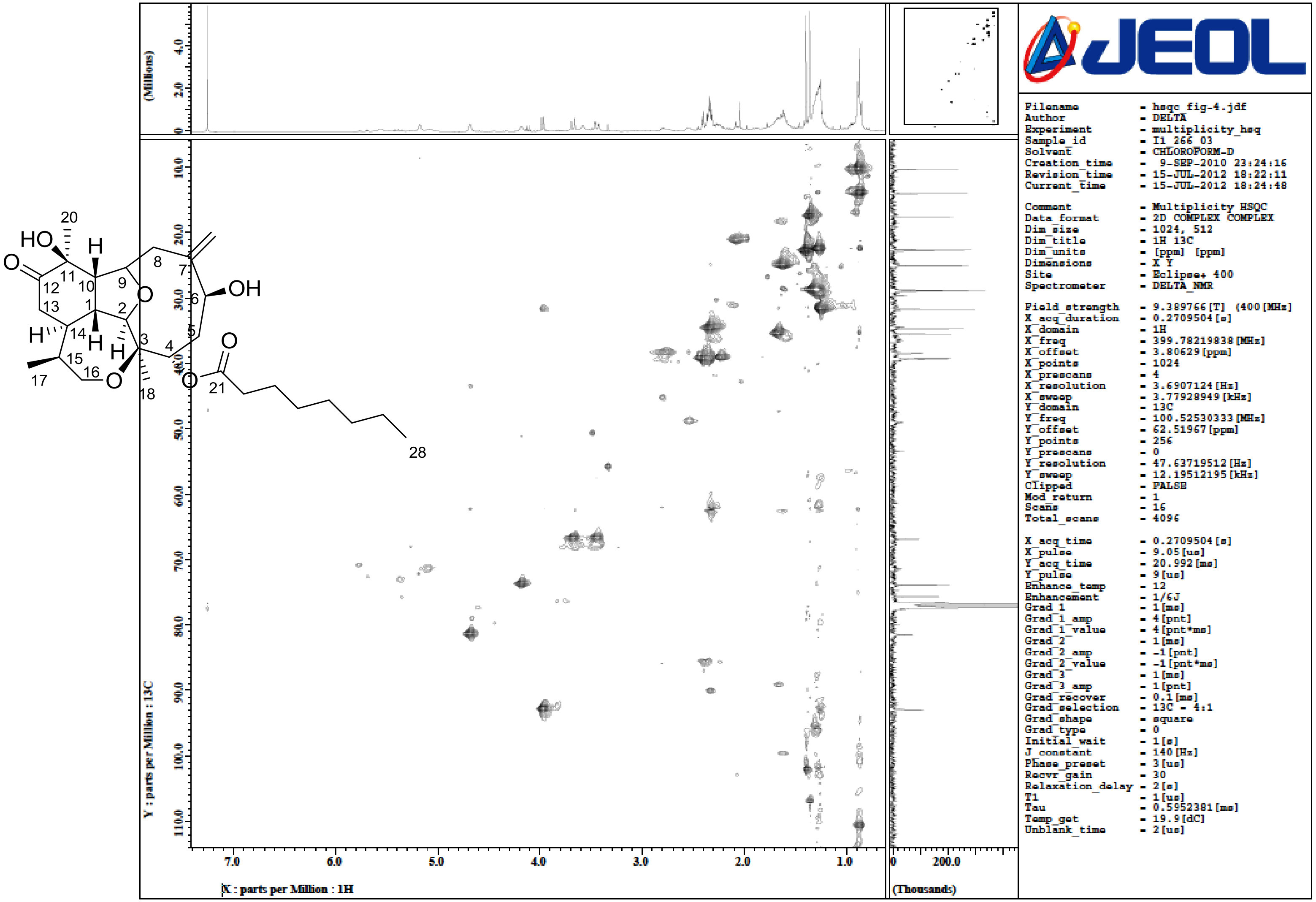

Figure S22. Briarellin S, HMBC spectrum.

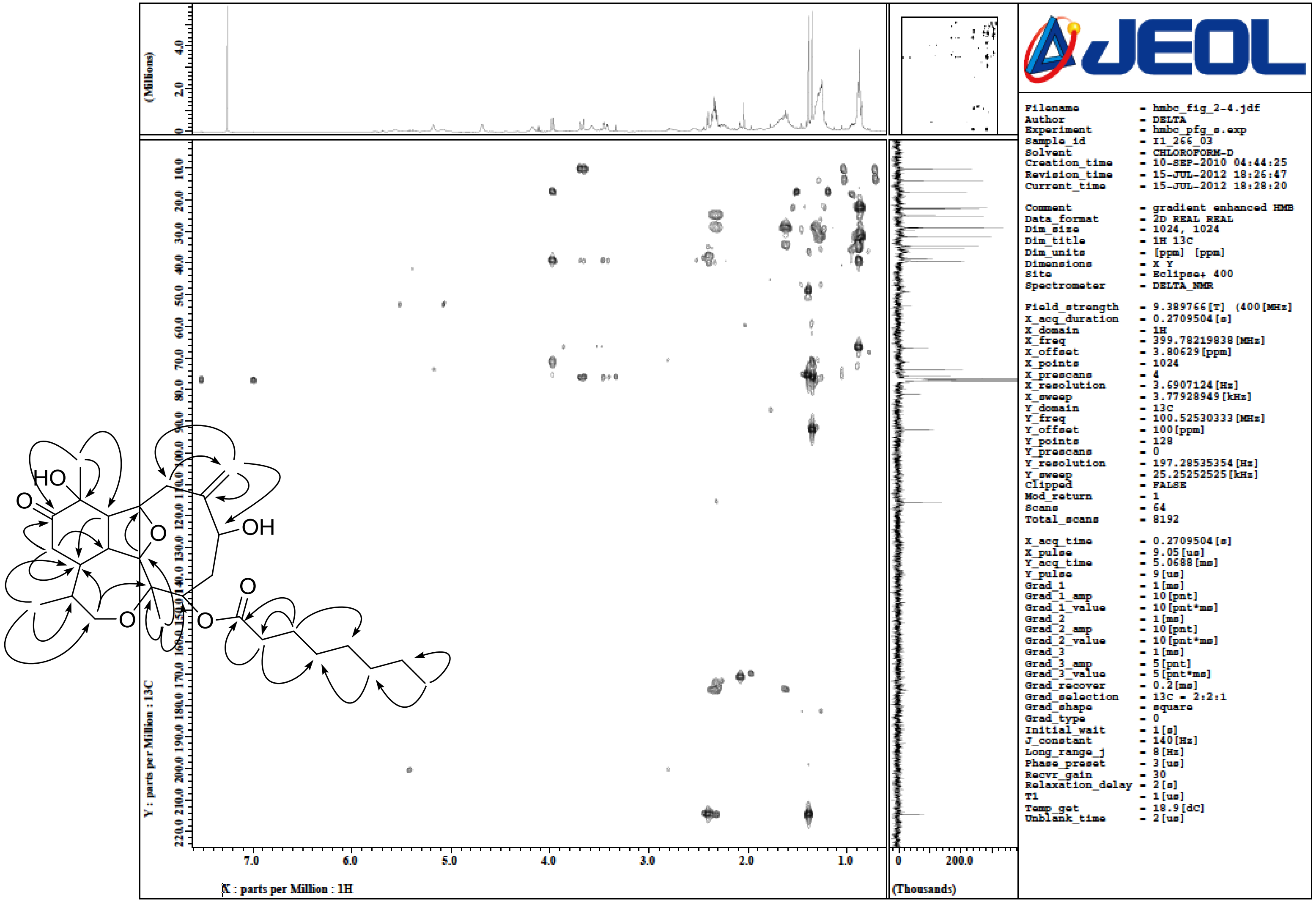

Figure S23. Briarellin S, selective DPFGSE-NOE spectrum by irradiation of H-4 and H-19a.

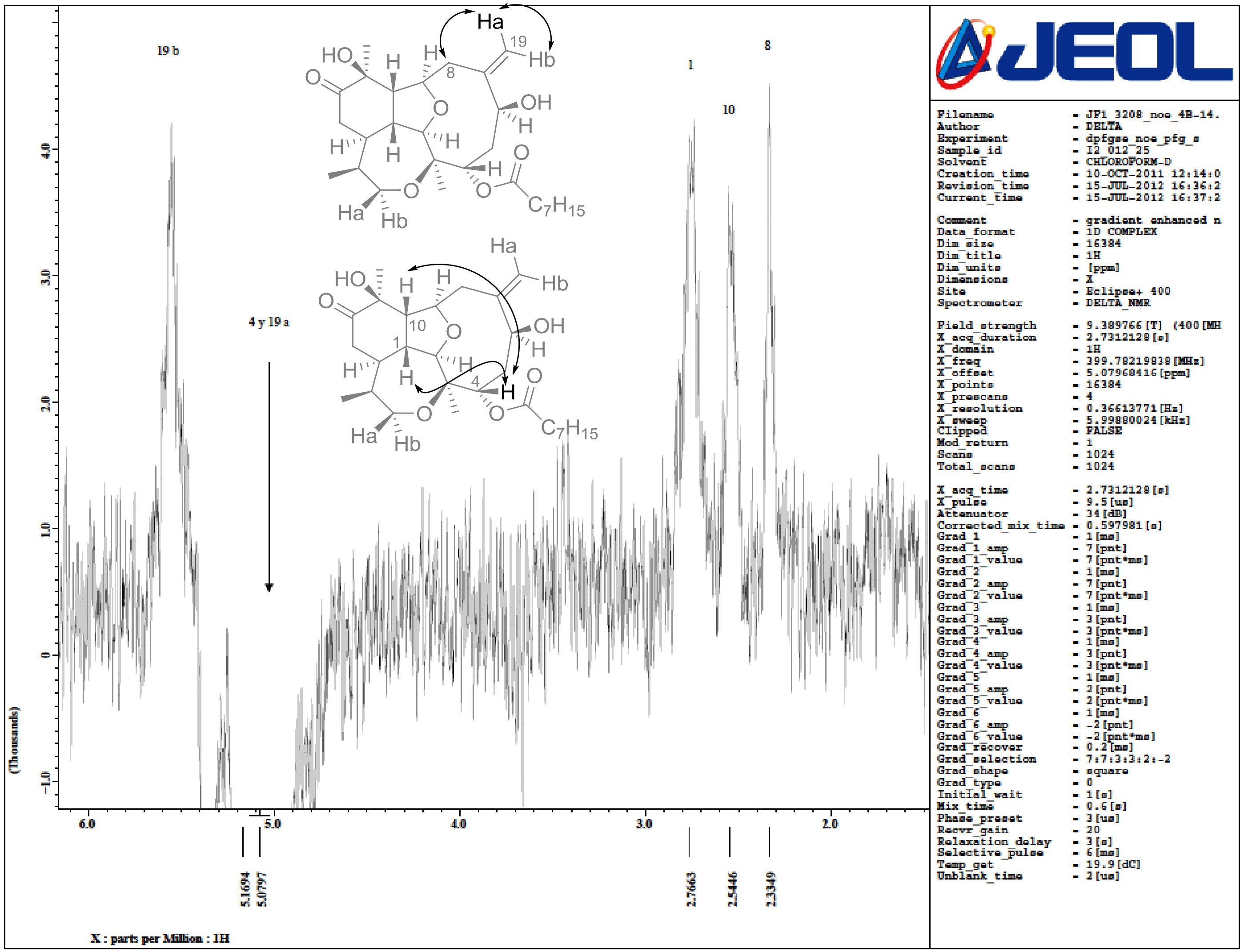

Figure S24. Briarellin S, selective DPFGSE-NOE spectrum by irradiation of H-6.

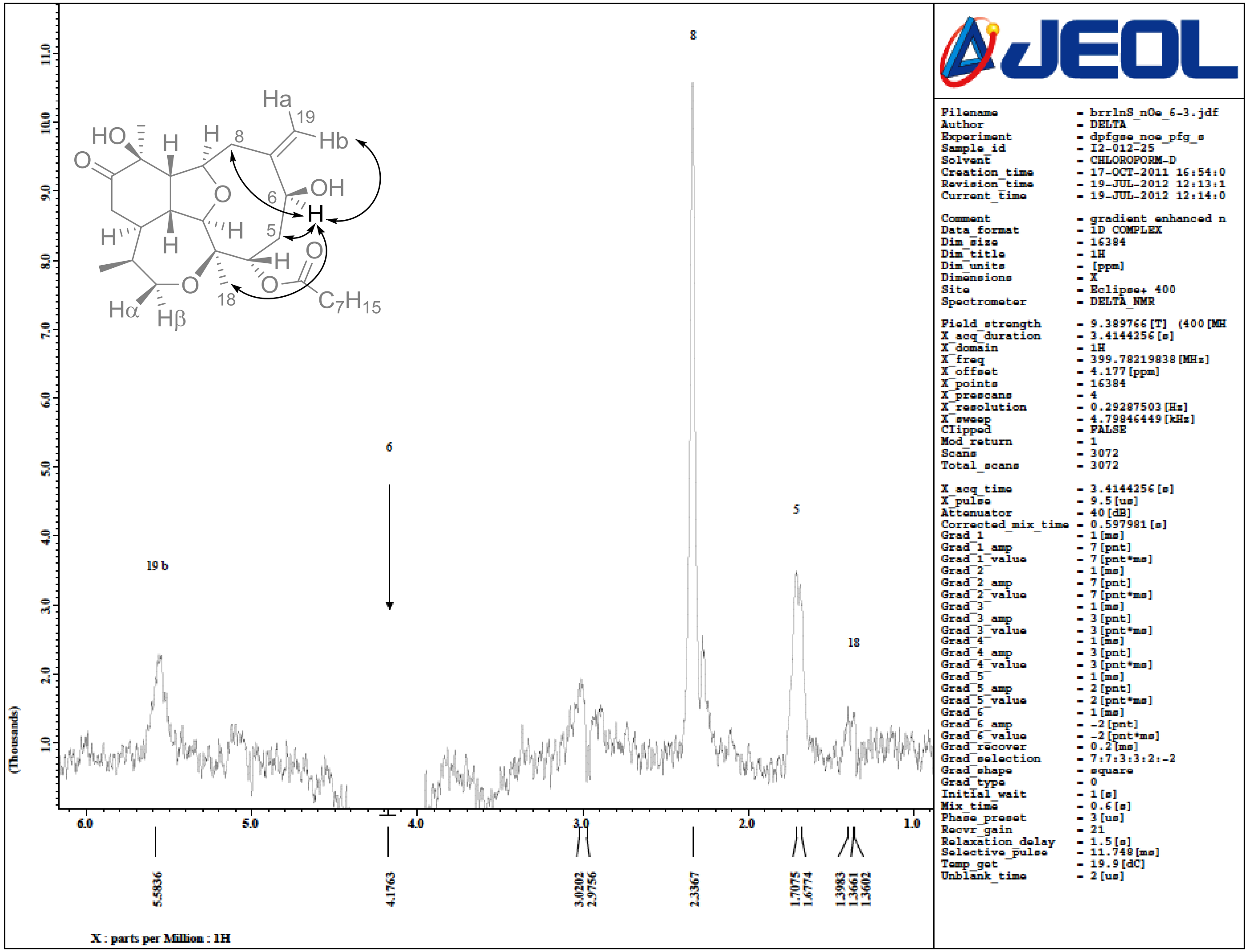

Figure S25. Briarellin S, selective DPFGSE-NOE spectrum by irradiation of H-17 and H-28.

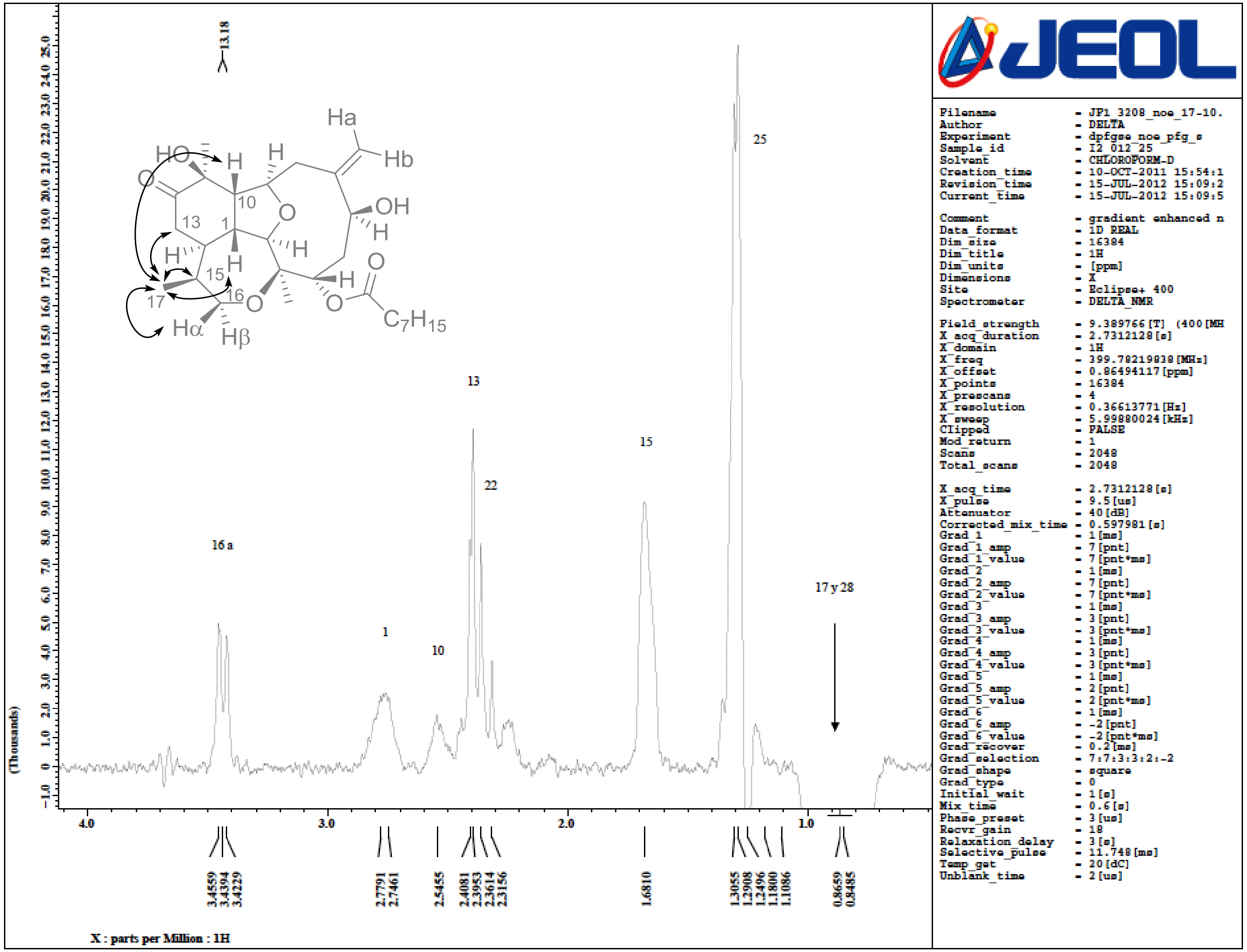

Figure S26. Briarellin S, selective DPFGSE-NOE spectrum by irradiation of H-18.

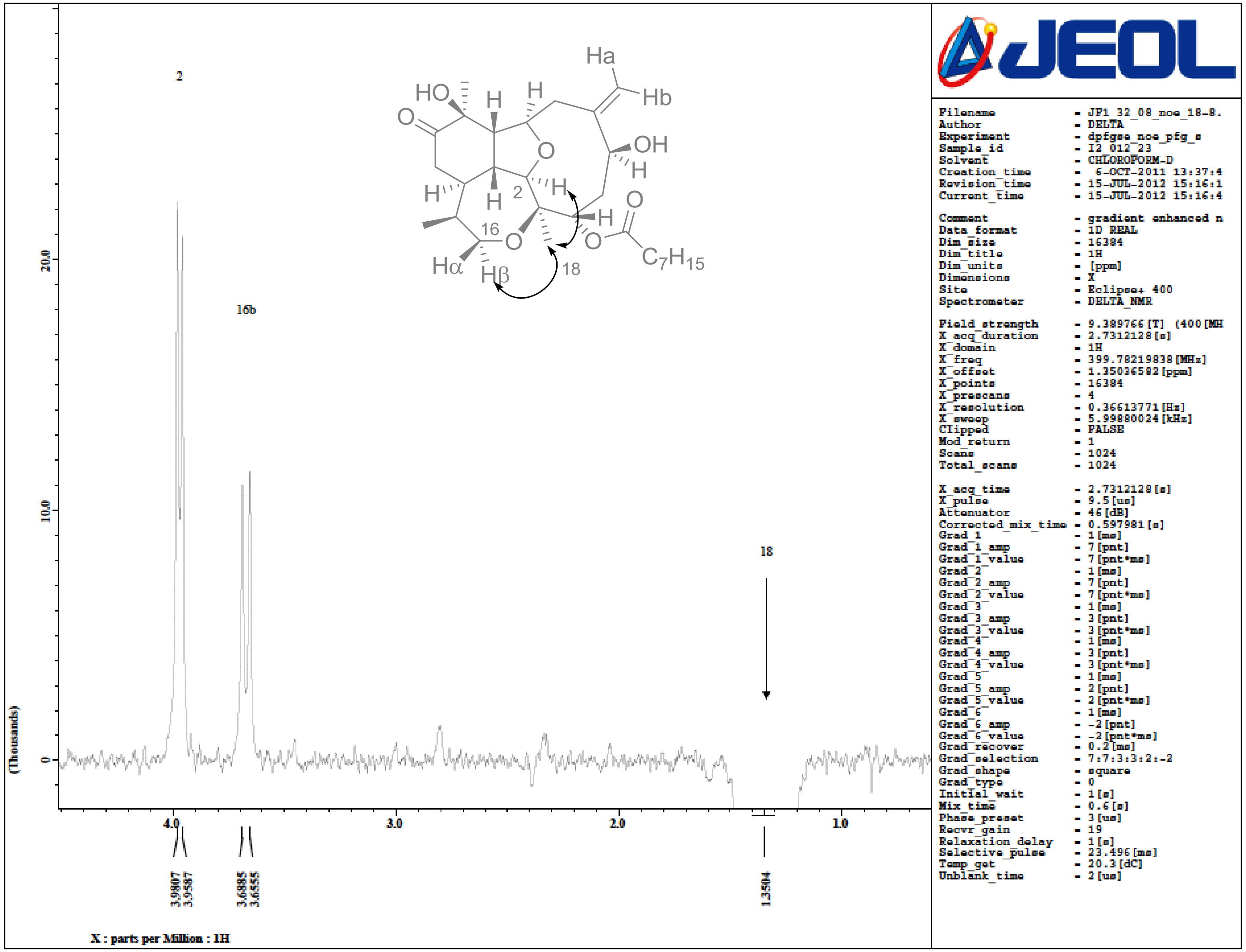

Figure S27. Briarellin S, selective DPFGSE-NOE spectrum by irradiation of H-20.

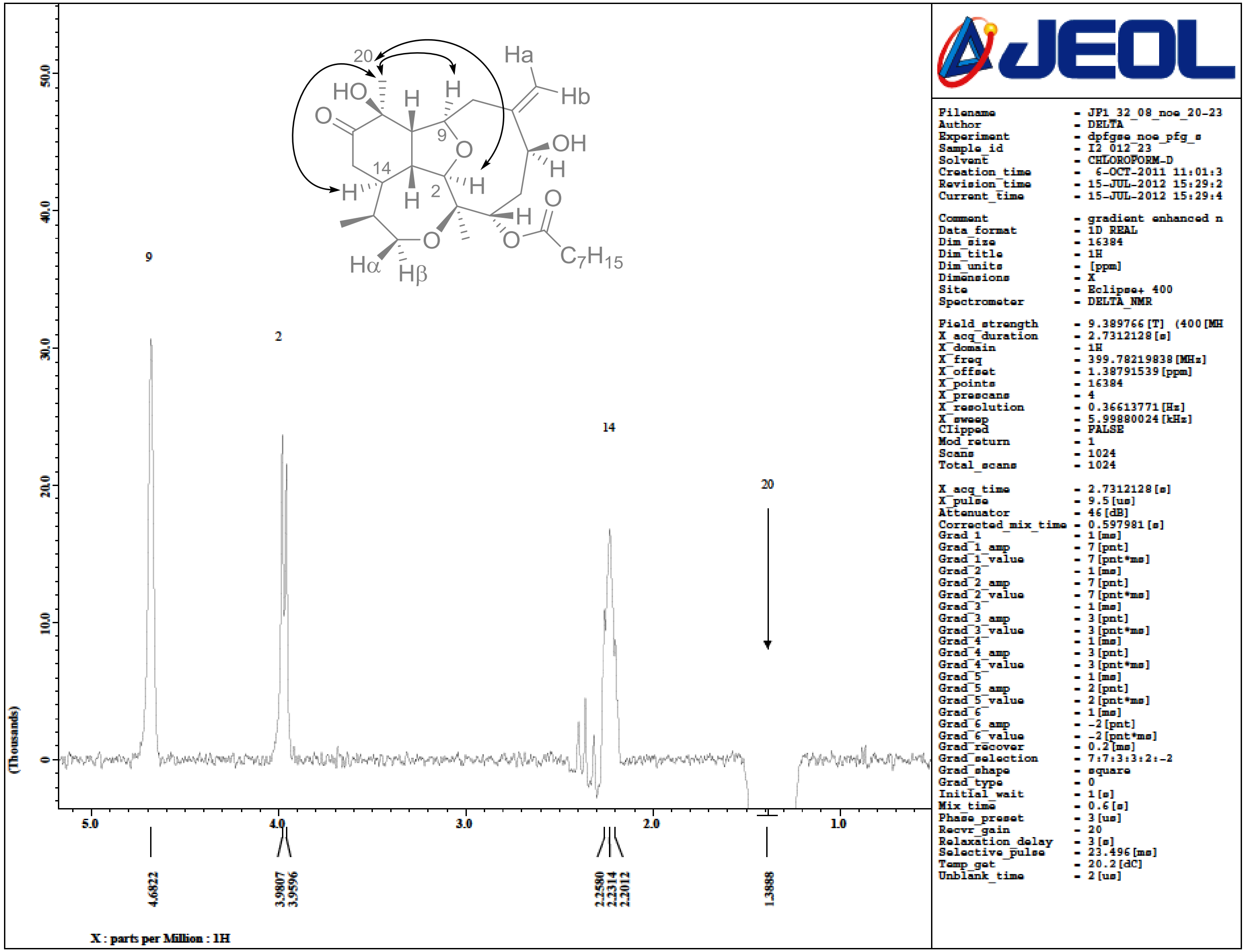

Figure S28. Briarellin S, HRMS-ESI-TOF spectrum. HSQC and HMBC correlations of the olefinic protons at C-19.

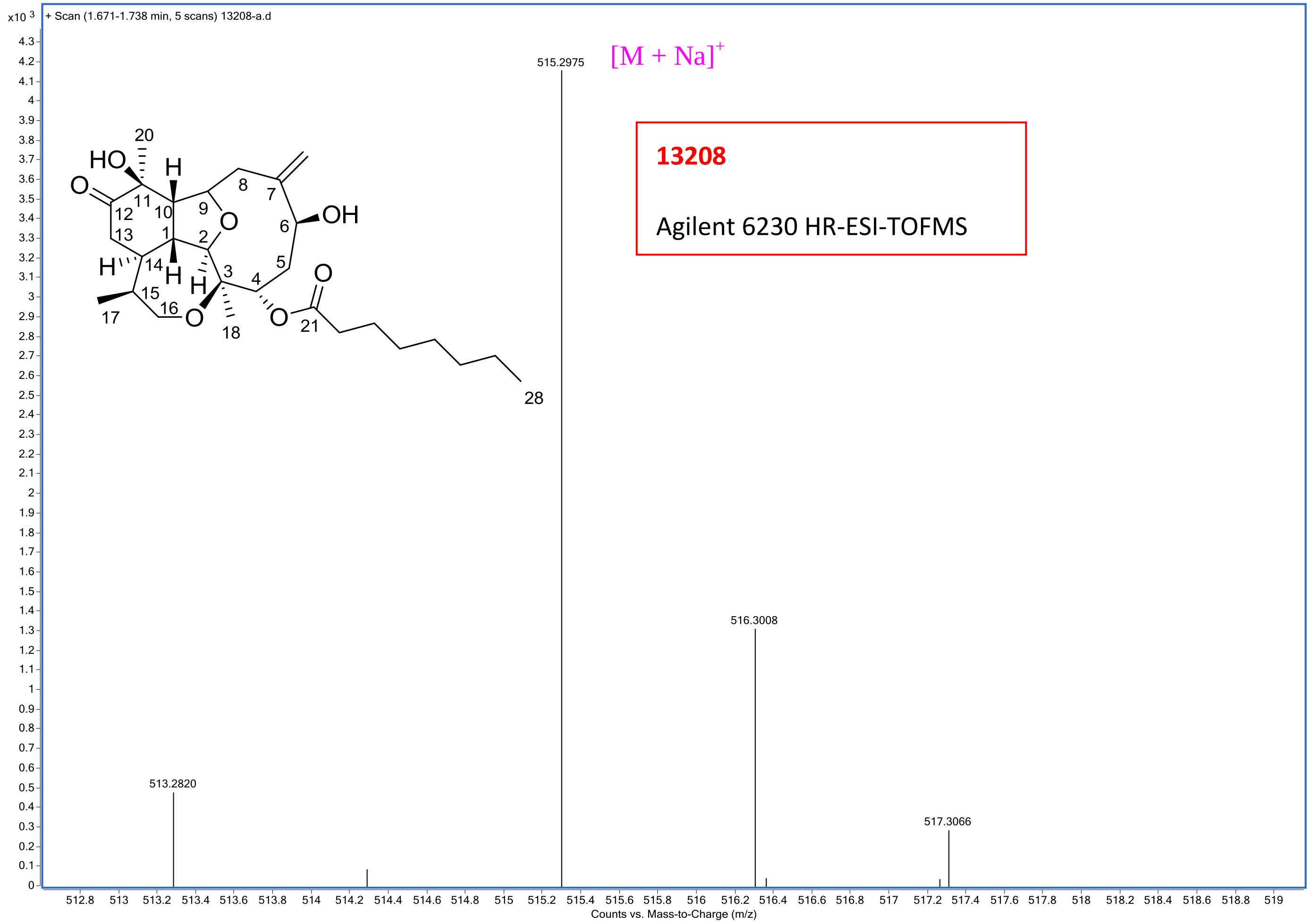

Figure S29. Briarellin S, HSQC correlation of H-19 (HSQC spectrum with a low peak threshold level).

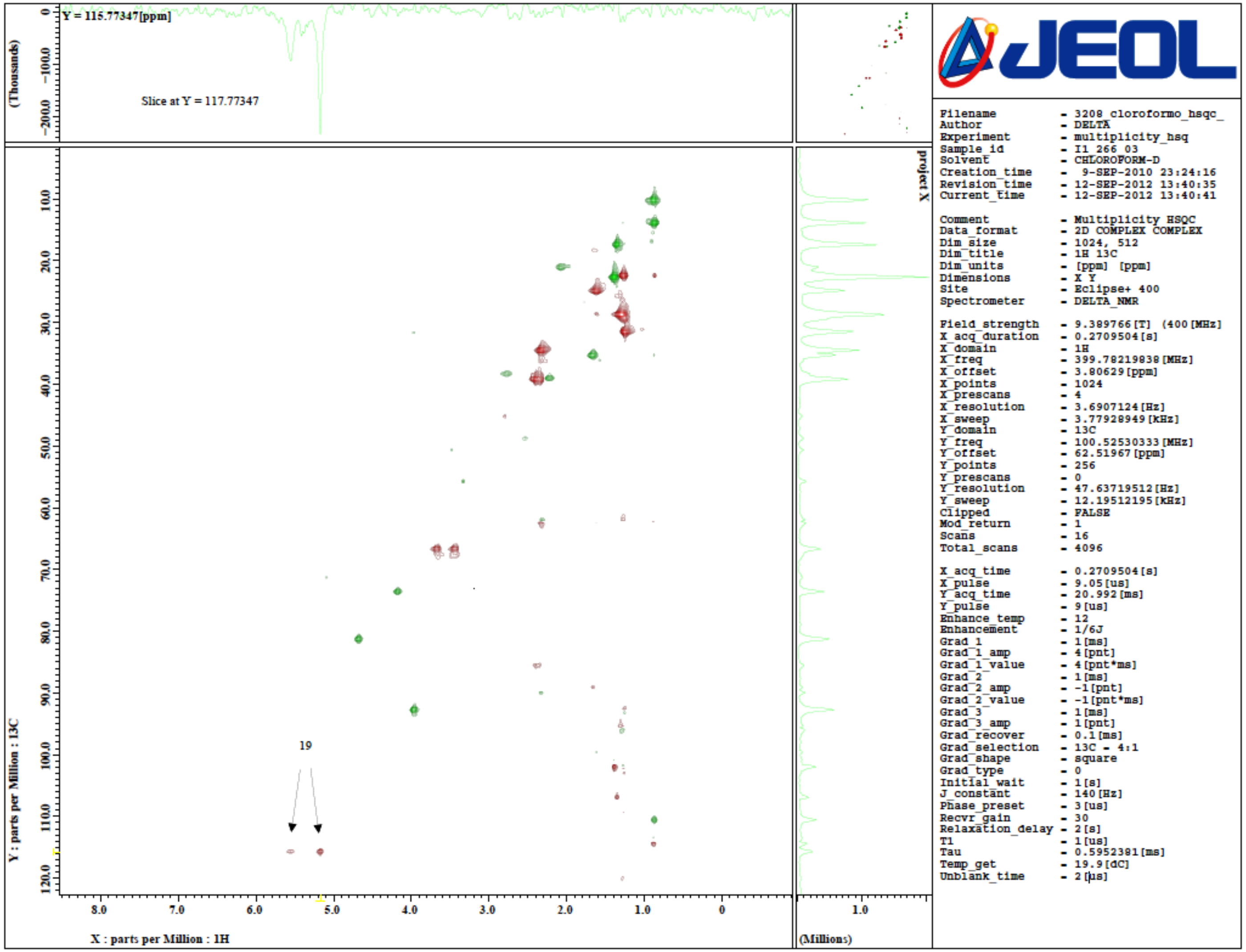

Figure S30. Briarellin S, HMBC correlations of H-19 (HMBC spectrum with a low peak threshold level).

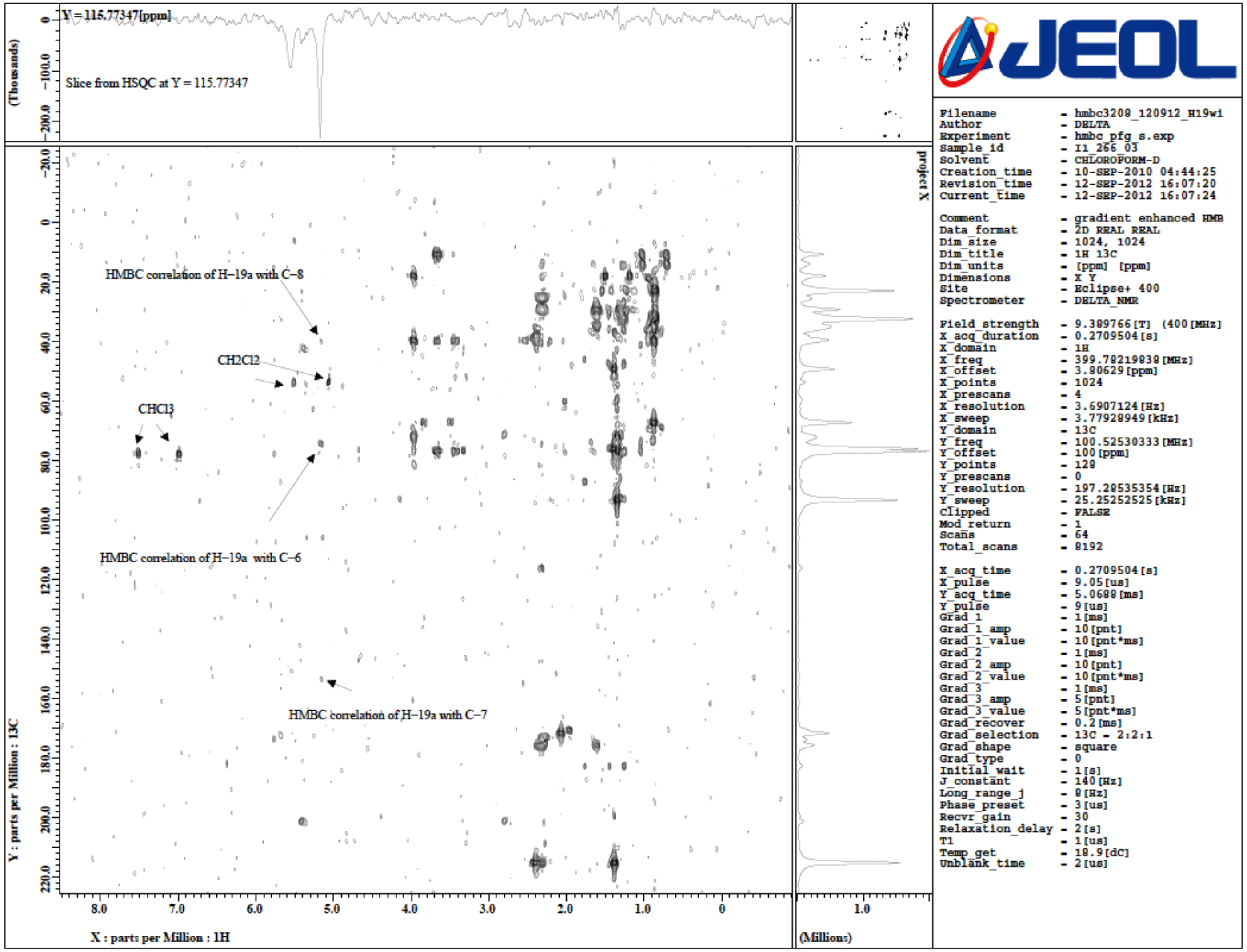

Figure S31. Briarellin S, HMBC correlations of H-19 (slice at *X* = 5.16).

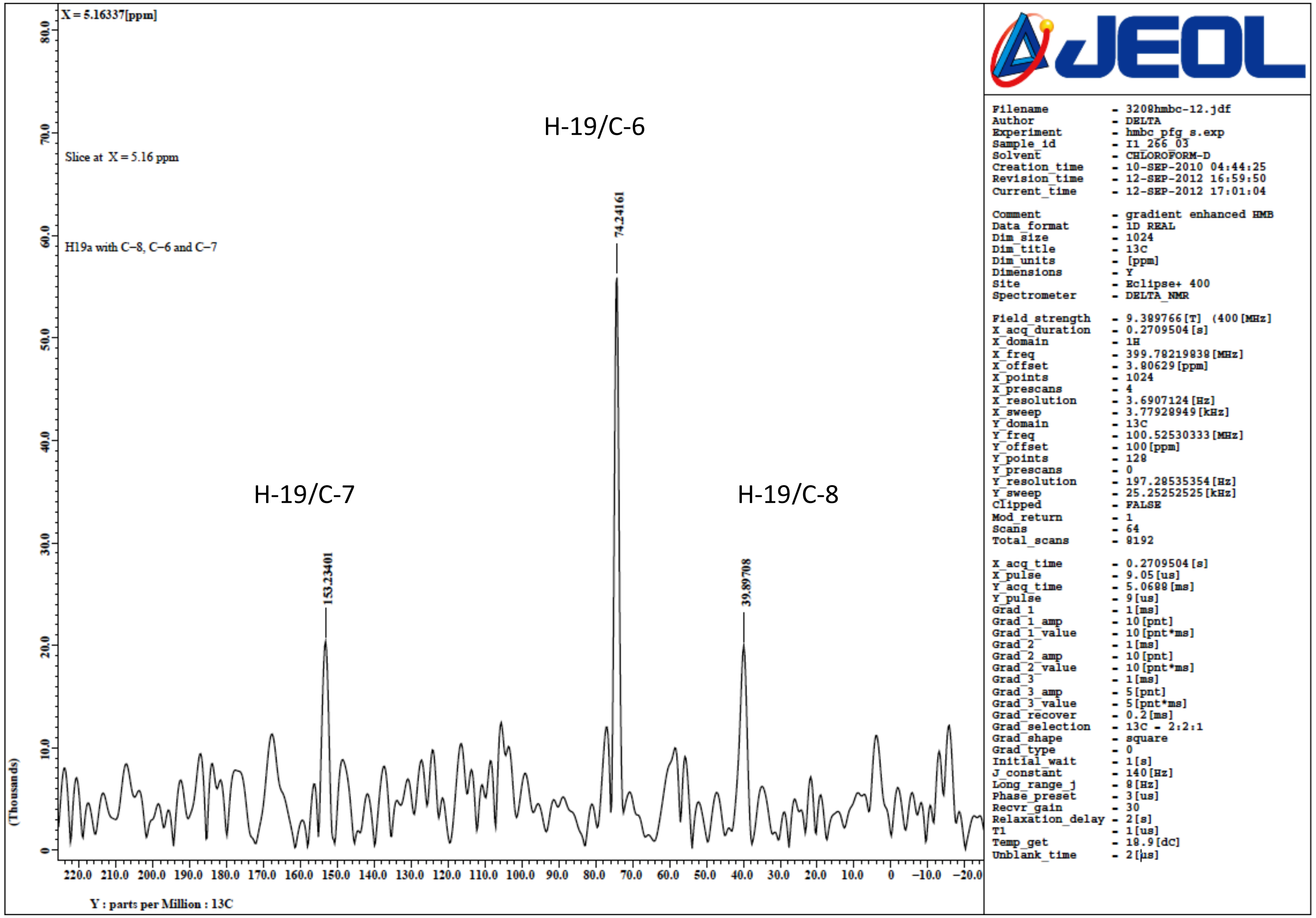

Figure S32. Briarellin S, HMBC correlations of H-19 (slices at *Y* = 39.90, *Y* = 74.49 and *Y* = 153.23).

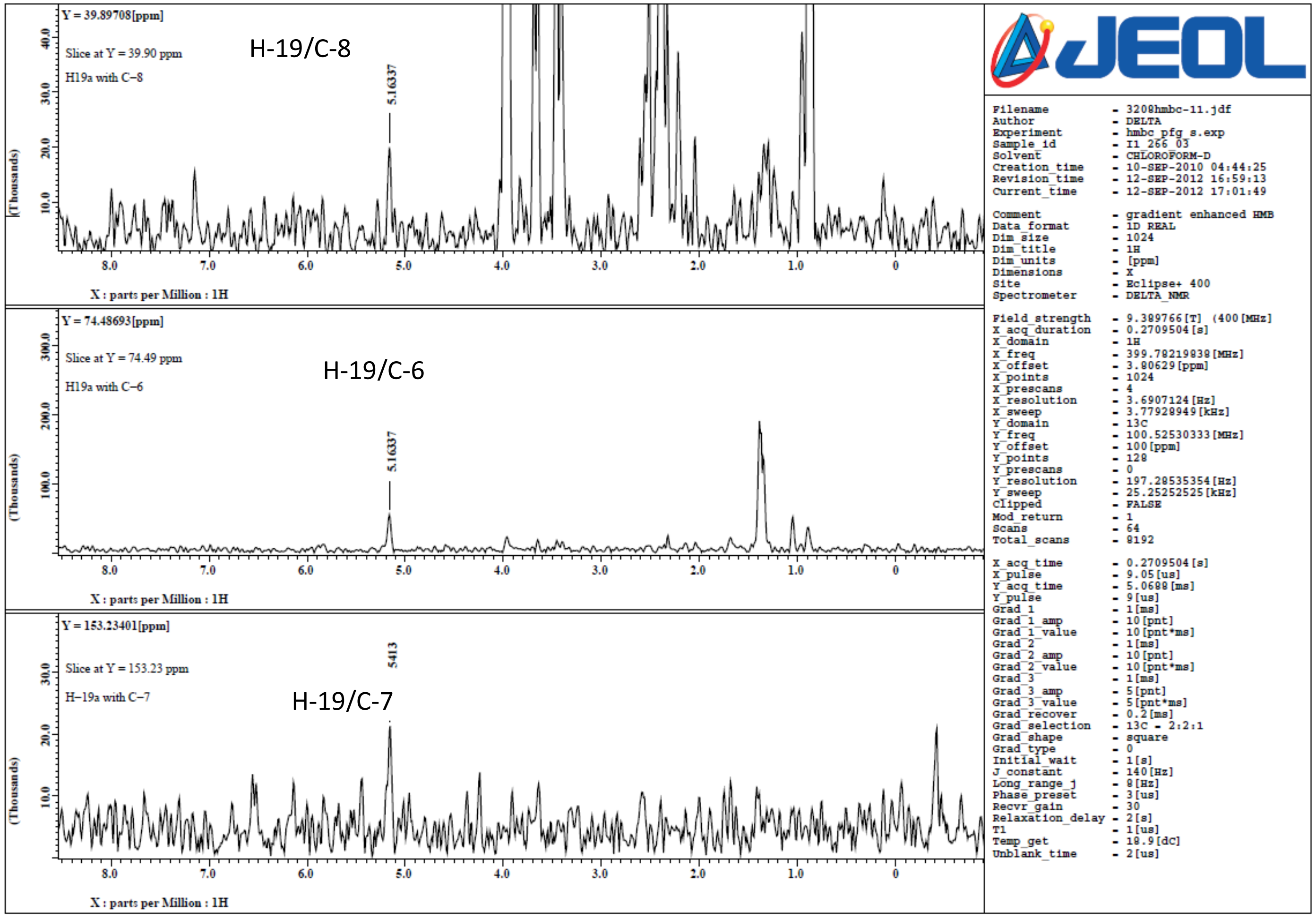

Supplement: Supplementary File 1: — Supplementary Information (PDF, 2942 KB) [file marinedrugs-10-02608-s001.pdf]
